# Therapeutic Potential of Sea Cucumber-Derived Bioactives in the Prevention and Management of Brain-Related Disorders: A Comprehensive Review

**DOI:** 10.3390/md23080310

**Published:** 2025-07-30

**Authors:** Purnima Rani Debi, Hrishika Barua, Mirja Kaizer Ahmmed, Shuva Bhowmik

**Affiliations:** 1 Department of Fish Biology and Biotechnology, Faculty of Fisheries, Chattogram Veterinary and Animal Sciences University, Chattogram 4225, Bangladesh; purnimacvasu@gmail.com; 2 Department of Fishing and Post-Harvest Technology, Faculty of Fisheries, Chattogram Veterinary and Animal Sciences University, Chattogram 4225, Bangladesh; hrishika.barua887@student.cvasu.ac.bd; 3 Department of Wine, Food and Molecular Biosciences, Lincoln University, Lincoln 7647, New Zealand; 4 Department of Fisheries and Marine Science, Noakhali Science and Technology University, Noakhali 3814, Bangladesh; 5 Centre for Bioengineering and Nanomedicine, Department of Oral Rehabilitation, Faculty of Dentistry, Division of Health Sciences, University of Otago, P.O. Box 56, Dunedin 9054, New Zealand

**Keywords:** Alzheimer’s disease, Parkinson’s disease, PI3K/Akt/GSK3β pathway, saponin, phenolic compounds, cerebrosides, glucocerebrosides

## Abstract

The popularity of bioactive compounds extracted from sea cucumbers is growing due to their wide application in the pharmaceutical industry, particularly in the development of drugs for neurological disorders. Different types of compounds, such as saponins, phenolic compounds, cerebrosides, and glucocerebrosides, are being studied intensively for their efficacy in assessing the treatment of neurodegenerative diseases, including Alzheimer’s disease, Parkinson’s disease, and brain tumors, among others. Positive results have been observed in the upregulation in the content of p-CREB, p-PL3K, BDNF, SOD, and MDA. Furthermore, the neuroprotective mechanism of the compounds against Alzheimer’s disease revealed that suppressing the phosphorylation of tau protein by the PI3K/Akt/GSK3β pathway leads to improved synaptic plasticity and reduced nerve fiber tangles. This comprehensive review explores recent findings on the therapeutic potential of sea cucumber bioactives in the treatment of brain-related disorders.

## 1. Introduction

Neurodegeneration is a progressive dysfunction and loss of neuronal structure and function that results in neuronal cell death. It is linked to deficiencies in particular brain processes (memory, mobility, and cognition), which can occur in several disorders affecting the central nervous system (CNS) [1,2,3,4]. The global prevalence of neurodegenerative diseases (NDs) such as Alzheimer’s disease (AD), Parkinson’s disease (PD), stroke, and depression has witnessed a significant rise, making them a serious health threat due to their chronic nature and severity [5]. As a result, these diseases have emerged as a research hotspot in biomedical science, particularly in the pursuit of effective therapeutic approaches. New research shows that a person’s genetic composition and environmental factors can significantly raise their risk of developing NDs [6], whereas age is the single biggest risk factor for the development of all NDs [7].

Although NDs differ clinically, there is a basic pathological mechanism for the occurrence of such disorders, including aberrant protein deposition, intracellular calcium (Ca^2+^) overload, mitochondrial dysfunction, imbalanced redox homeostasis, and neuroinflammation [8,9]. The main contributing factor is the accumulation and deposition of toxic proteins in the brain, and mitochondrial dysfunction is an important variable in the progression of the disease [6,10]. Previous studies have indicated that the survival rate of NDs is limited [11,12,13]. A current investigation in 2019 stated that there were 349.2 million people affected by severe neurological illnesses and 10 million fatalities globally [14,15]. Among all these medical conditions, AD was the leading cause of mortality, next to cognitive impairment and neonatal dementia [16]. The number of fatalities has risen by 39% in absolute terms during the last 30 years, and the number of disability-adjusted life-years has increased by 15% [17].

Depending entirely on the disease’s nature and stage, these disorders may have a variety of neuropathological traits that can be extremely dangerous or even fatal in some cases. Currently, several management strategies are recognized which either focus on the pathophysiology of the disease or try to alleviate its symptoms [6]. However, the rate at which these diseases are becoming more common is outpacing the rate at which they can be managed and treated. While several neuroprotective and disease-modifying drugs have shown significant promise in preclinical research, finding novel chemical compounds to serve as design templates for drug development initiatives is becoming a growing challenge [18]. This initiates the need for the exploration of natural bioactive compounds to treat disease progression and also to achieve a complete remission.

Since ancient times, natural products have been used and recognized for their healing properties. The protective effects of natural products and the isolated bioactive components against various diseases, including cancer, diabetes, heart disease, reproductive disorders, and neurological diseases, have been the subject of numerous studies in recent decades [19,20]. In the case of neurodegenerative diseases, the mode of action may involve multiple mechanisms, including antioxidant, anti-inflammatory, and antiapoptotic activities. Due to the diverse spectrum of pharmacological and biological actions, natural products are seen as potential alternatives for the treatment of neurodegeneration, aiding in the creation and discovery of new medications [21,22]. Currently, in addition to terrestrial sources of natural products, marine organisms such as microorganisms, vertebrates, invertebrates, and algae are gaining prominence in the extraction of pharmacologically active substances as a research interest [23]. Given the growing burden of infections, metabolic abnormalities, and age- and lifestyle-associated diseases, there is a pressing need for ongoing exploration of marine bioactives through both conventional and contemporary approaches [24]. Numerous chemical substances like bioactive peptides, fatty acids, pigments, alkaloids, and polysaccharides have previously been identified from marine sources and could be beneficial in preventing and treating a range of neuroinflammatory diseases [25]. These findings have brought the sea cucumber, a marine organism, into the spotlight of scientific circles due to its rich nutritional content and potential to support neurological health [26].

Among different valuable marine resources, sea cucumbers are one of the most promising ones, belonging to the class Holothuroidea. These organisms are soft-bodied marine invertebrates with leathery skin and an elongated shape with a single, branched gonad. There are 1716 species of sea cucumbers, with the Asia–Pacific region having the highest biodiversity [27]. Due to the exposure to the challenging and dynamic environment, sea cucumbers have evolved to produce distinct secondary metabolites with biological activity [28]. Sea cucumbers have been used for their medicinal properties since ancient times. According to the Ming dynasty account (1368–1644), sea cucumbers were known as “haishen,” or “ocean ginseng,” since they had the same medical qualities as ginseng [28]. Within the framework of Traditional Chinese Medicine, sea cucumbers have been utilized as a revitalizing agent to treat skeletal and joint weakness, especially that associated with age-related inflexibility, renal system abnormalities, sexual disorders, dry-stool constipation, poor lipid digestion, and circulatory complications [29].

However, the creation and discovery of novel medications greatly benefit from their taxonomic variety, diverse biological activity, high production, and chemical distinctiveness [30,31]. The active compounds isolated from these organisms exhibit broad chemical diversity, including polysaccharides like glycosaminoglycans (mucopolysaccharides), neutral glycans, fucosylated chondroitin sulfates (FCSs), and sulfated fucans, as well as peptides, phospholipids, and glycolipids, including glycosphingolipids (cerebrosides), polyunsaturated fatty acids, phenols, and triterpene glycosides (saponins) [32,33,34,35,36,37,38]. For example, FCS is a distinct type of sulfated glycosaminoglycan that is only present in echinoderm sea cucumbers, which differs from other known mammalian glycosaminoglycans in both structure and function [39]. One of the most unique features of sea cucumbers is their ability to regenerate internal organs and body parts which is far higher than that of sea stars and sea urchins, making them excellent models for regeneration [40]. For instance, the presence of a unique genomic profile related to intestinal regeneration in *Apostichopus japonicus* holds promising biomedical implications [41]. These tissue-healing capabilities may play a role in hindering tumorigenesis by preventing the transformation of epithelial cells into cancerous cells [42].

**Figure 1:**
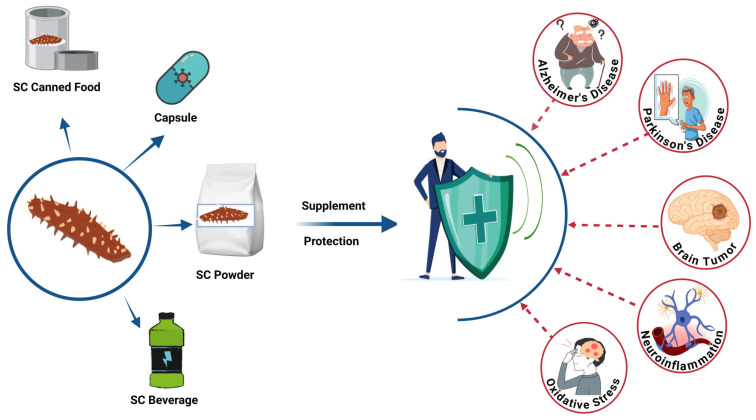
Potential neuroprotective applications of sea cucumber-based products. SC, sea cucumber. The figure was generated from the concept of Man et al. (2022) [45] using Biorender.com (Agreement number: FF28KHIJ6E).

The neuroprotection potency of sea cucumbers’ bioactives (SCBs) was shown in several animal models [43,44]. Thus, this review aims to examine evidence of potential therapeutic bioactives derived from sea cucumber for the prevention and management of brain-related disorders such as Alzheimer’s disease, Parkinson’s disease, brain tumors, neuroinflammation, and oxidative-stress modulated brain disorders (Figure 1).

Sea cucumber (SC)-derived products, including canned food, capsules, powders, and beverages, can be formulated as dietary supplements for brain health [35,45,46,47,48]. These bioactive-rich formulations may provide protection against various brain-related disorders through their multifunctional therapeutic properties [45,47]. While research on the development of SCBs is attracting more attention, the ability to translate these new findings into industrial practice and new products remains immature. Challenges, including validating health claims, unclarified mechanisms, unsatisfactory sensory properties, and high production costs, must be solved. This review provides a comprehensive and up-to-date overview of the biological functions of SCBs and discusses potential solutions to the identified issues. This information will provide a general perspective on developing SCB nutraceuticals and functional foods.

## 2. Research Methodology

In order to search the relevant published articles, multiple search engines such as Scopus, PubMed, Web of Science, and Google Scholar were used. The search strategy was focused only on English-language scientific articles published between 2000 and 2025. More than 100 keywords containing one- to seven-word phrases like “toxic protein deposition in the brain”, “alpha-synuclein (α-Syn) aggregates”, etc., were used (Table 1).

**Figure 2:**
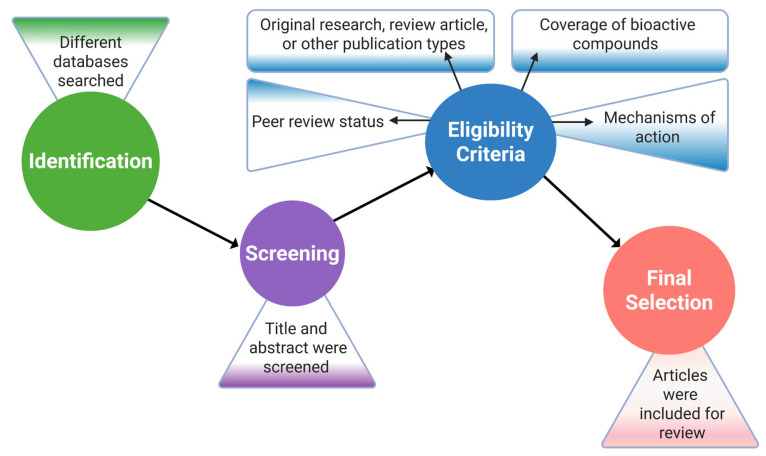
Research article selection process flowchart. This figure was generated using Biorender.com (Agreement No: MX28J5UCP2).

The article selection process was based on important criteria, for example, the peer review status, the type of publication, including original research, review article, or other publication types, the coverage of bioactive compounds extracted from the target organism, and the effects as well as the mechanisms of action of the compounds in the body. Following that, the selected articles were further assessed based on the study objectives, appropriateness of the methods, accuracy of the data analysis, and effectiveness of the findings [49]. In order to increase the credibility and coherence of the results by finding parallels and differences among studies, a meticulous cross-referencing and validation process was employed, and studies with inconsistent results or a poor methodology were omitted (Figure 2).

## 3. Bioactive Compounds in Sea Cucumbers

**Figure 3:**
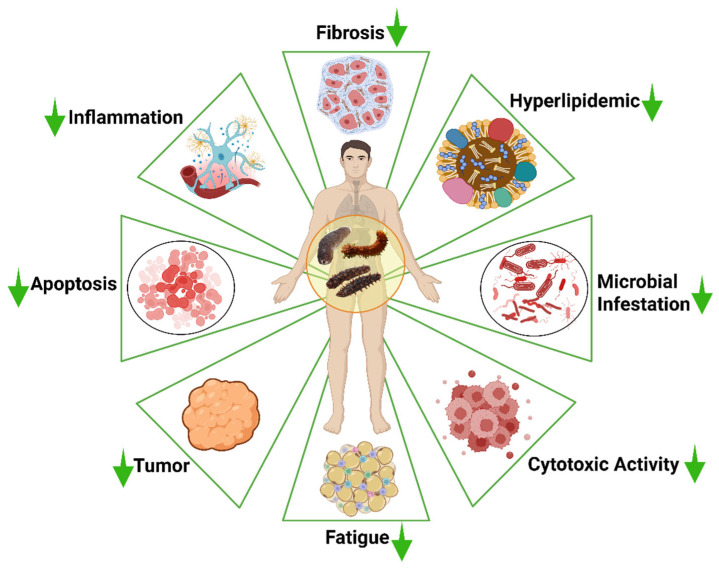
Bioactive compounds derived from sea cucumbers and their associated overall pharmacological properties. The concept of the figure was adopted based on the general approach described by Salindeho et al. (2022) [50] using Biorender.com (Agreement number: MQ28J0DC2Z).

Most of the body parts of the sea cucumber, including the processing discards, which account for over 50% of the body weight of sea cucumbers [37], may contain a wide range of bioactive compounds. These bioactive compounds may include peptides, phenols, triterpene glycosides, fucoidan, fucosylated chondroitin sulfate (FCS), cerebrosides, and sphingoids. These bioactive compounds extracted from sea cucumbers have multifunctional therapeutic potential in biomedical and nutraceutical applications, such as anti-cancer, antihypertensive, antioxidant, antidiabetic, anti-tumor, anti-inflammatory, anti-microbial, and wound healing properties (Figure 3).

### 3.1. Saponin

Sea cucumbers are among the few animal lineages that produce saponins, secondary metabolites, which are widely found in plants [51]. In contrast to the typical creation of lanosterol in animal cholesterol synthesis, Li et al. (2018) noted that the sea cucumber has “plant-like” patterns that are characteristic of evolutionary convergence, which enable it to produce parkeol for saponin synthesis through oxidosqualene cyclase [52]. The molecular structure of sea cucumber saponins is typically a triterpenoid oligoglycoside, joined by sugar chains via the β-glycosidic link and a non-polar (fat-soluble) aglycone. Based on the carbon structure of the non-polar aglycone region, these glycosylated molecules, also known as glycosides, are classified into three primary groups: triterpenoidal glycosides, steroidal glycosides, and steroidal alkaloid glycosides [42,53,54]. The majority of sea cucumber saponins exist in the holostane form; however, they are frequently separated into holostane and nonholostane forms based on the varying positions of the aglycone lactones [55]. Currently, several newer saponins can be isolated and characterized from sea cucumber through techniques like liquid–liquid extraction with various solvents, Soxhlet extraction with 70% solvents, solid phase chromatography with silica gel or resins, etc. [56]. Apart from these, different advanced methods are used in the extraction, optimization, and characterization of these bioactive compounds like high-performance liquid chromatography (HPLC), time-of-flight mass spectrometry (TOF/MS), matrix-assisted laser desorption ionization–time-of-flight mass spectrometry (MALDI-TOF MS), and high-performance centrifugal partition chromatography (HPCPC), high-resolution mass spectrometry (HRMS), and nuclear magnetic resonance (NMR) [56,57,58]. Numerous sea cucumbers and their various body parts have been identified to contain a variety of saponins, including Frondoside A, Echinoside A, Cucumarioside A2-2, Holotoxin A1, Stichoposide C, Acetylated Lessoniosides A-E, and Non-acetylated Lessoniosides F and G [59,60], and there is a correlation between the molecular structure and bioavailability of these compounds. For instance, in the rat model, Echinoside A, with a lower molecular mass and less complex glycan structure, exhibited higher bioavailability than Holotoxin A1, likely due to reduced sterically branched chains [61].

Due to the potential of saponin for neuroprotective management and effects on the attenuation of diseases of the central nervous system, saponins accounted for 72% of sea cucumber research in the field of tumor and cancer management [60,62].

Saponin extracted from *Holothuria leucospilota* showed antioxidant activity in 2,2-Diphenyl-1-picrylhydrazyl (DPPH), 2,2′-azino-bis-(3-ethylbenzothiazoline-6-sulfonic acid (ABTS), and the reducing power assay, indicating that it can lower reactive oxygen species (ROS), a major contributor to brain stress. Additionally, another study found that the extract activates the daf-16/Forkhead box O (FOXO) pathway in Caenorhabditis worms, hence mediating lifespan extension and stress tolerance [63,64]. Together with other bioactive substances from the body wall and Cuvierian tubule of *H. leucospilota*, saponin-rich extract improved dopaminergic (DA) neuronal function in food-sensing behavior and reduced α-synuclein aggregation in in vivo PD models [65]. In a study on a transgenic *C. elegans* AD model, the effects of frondoside A from sea cucumber (*C. frondosa*), a saponin, on amyloid-beta (Aβ) aggregation and proteotoxicity were assessed, in which frondoside A considerably postponed the worm paralysis brought on by Aβ aggregation and restored chemotaxis failure in worms whose neurones produce Aβ, and shielded the worms from oxidative stress [66].

### 3.2. Peptides and Proteins

Biologically active peptides are parts of naturally occurring proteins that are inactive in their precursor form but exert a physiological effect upon enzymatic release or transport to the active site [67]. They are generally a group of peptides, in most cases consisting of fewer than 50 residues, that have a function in a living organism or cell. Although some of these peptides are found in a bare format, many of them are hidden in the intact structure of protein molecules [68]. Since they have nutraceutical potential, these protein hydrolysates and peptide fractions are widely used as functional food additives in the pharmaceutical industry [69]. As such, their widespread potential has led to their increasing application in disease prevention and quality health promotion, while growing scientific and commercial interest [70].

There are several techniques for synthesizing bioactive peptides from natural sources, which fall into three categories: in vitro, in vivo, and in silico. To create bioactive peptides, particularly commercial enzymes, in vitro techniques, including microbial fermentation, chemical hydrolysis, and enzymatic hydrolysis, are frequently employed. Enzymatic hydrolysis is considered the crucial step for sea cucumber peptides (SCP) production, where a variety of parameters influence the ultimate yield and bioactivity of SCP. Hence, a majority of research on SCP has used enzymatic hydrolysis techniques [45]. In the majority of studies, commercial food-grade enzymes such as Trypsin, Flavorzyme, and Alcalase were used to ensure a better yield of peptides, due to their better efficiency and operational suitability, as the resulting peptides demonstrated potent antioxidative activity [71,72,73,74].

Bioactive peptides isolated from the sea cucumber show antioxidative and neuroprotective properties by targeting various mechanisms such as reduced ROS production, enhanced acetylcholinesterase (AChE) activity in mouse brains, regulating acetylcholine (Ach) and AChE activity to protect the cholinergic system, and choline acetyltransferase (ChAT) upregulation, which is associated with improved memory performance [73,75,76]. Furthermore, neurodegenerative illnesses can be improved by sea cucumber-derived biopeptides or enzymatic hydrolysates in many ways, such as preserving the redox balance, reducing mitophagy, boosting cell survival, encouraging neuron organization and form, and controlling the cholinergic system. In silico bioinformatics analysis revealed that SCPs were found to be ACE inhibitory, antioxidative, and most importantly, demonstrated the structural attributes including hydrophobicity, low molecular size, and amino acid (AA) composition, which addresses their strong correlation with absorption, distribution, metabolism and excretion (ADME) performance, and oral bioavailability, aligning closely with the pharmacokinetic properties of captopril [77]. It has been discovered that short peptides have a strong neuroprotective effect [78]. In a study, the effect of SCP extracted from *S. japonicus* on memory impairment was evaluated, where over 92% of short peptides enhanced synaptic plasticity and controlled dopamine/serotonin metabolization through the TH/VMAT2 pathway, and the possibility of blood–brain barrier (BBB) crossing was suggested [79].

Again, SCP shows antioxidant properties by compensating for glutathione depletion, lowering mitochondrial superoxide levels, reducing mitophagy, and protecting human neuroblastoma cells against hydrogen peroxide (H_2_O_2_) [80]. In H_2_O_2_-exposed Vero cells, Lee et al. (2021) discovered that αchymotrypsin-assisted biopeptides from sea cucumber (*S. japonicus*) significantly reduced intracellular ROS levels and deoxyribonucleic acid (DNA) damage, preserved cell integrity, and boosted cell viability [72]. Moreover, in vivo research showed that the peptide fraction of *S. variegatus* and *C. frondosa* effectively increased longevity in both normal and D-galactose-induced aging fruit flies and reduced oxidative damage in mice by upregulating Klotho expression, activating superoxide dismutase (SOD) and glutathione peroxidase (GSH-Px), and preventing protein oxidation and lipid peroxidation [81,82]. Apart from this, SCP from *A. leucoprocta* improved cognitive dysfunction in D-gal-induced aging mice. SCP activated a GABA_B_R/cAMP/PKA/CREB pathway (γ-aminobutyric acid type B receptors (GABA_B_R)/cyclic adenosine monophosphate (cAMP)/cAMP-dependent protein kinase A (PKA)/cAMP response element-binding protein (CREB)) that boosts the release of GABA (gamma-aminobutyric acid), a brain chemical that calms the nervous system [83].

### 3.3. Polysaccharides

Polysaccharides such as sulfated polysaccharides (fucosylated chondroitin sulfate, or FCS), sulfated fucan or fucoidan, non-sulfated polysaccharides, or neutral glucan are abundant in sea cucumbers, mostly in the body wall [33,84,85,86]. The sulfation pattern of the monosaccharide composition determines the bioactivity of FCS, an exclusive glycosaminoglycan [87]. Despite interspecific variations, all FCSs share a core backbone of {3)-D-GalNAc-(β1,4)-D-GlcA-(β1,}, with the branching regions differing in the sulfation pattern and glycosylation of fucosyl groups [33]. As opposed to branching FCSs, sulfated fucans from sea cucumbers are frequently linear polymers made up of repeating structural components [88,89,90]. A previous study found that compared to native FCS polysaccharides (~9%), sea cucumber-derived FCS oligomers demonstrated a greater absorption rate (~32%), leading to improved bioavailability [91].

In addition to their well-documented bioactivities, including anticoagulant, anti-cancer, antithrombotic, and antibacterial effects, sea cucumber-derived polysaccharides also exert neuroprotective properties, though the neuroprotective effects are influenced by the species-specific structural variations within the polysaccharide molecules. According to Li et al. (2020), *S. chloronotus* fucoidan mostly consists of L-fucose and sulfate esters, exhibiting immunoregulatory and lipid peroxidation inhibition [92]. Furthermore, the gonadal polysaccharide of sea cucumbers (*A. japonicus*) demonstrated reducing power, DPPH, and hydroxyl radical-scavenging properties, maintaining a lower molecular weight while having a larger sulfate group concentration increased the activity [93]. Li et al. (2021) reported that polysaccharides derived from the sea cucumber *C. frondosa* lessen the cytotoxicity and aggregation of Aβ40, one of the factors that causes AD [94]. Neural stem cell (NSC) is a strong contender for cell replacement treatment [95,96] for several untreatable CNS conditions. In a study, Cui et al. (2016) found that a polysaccharide from *S. japonicus* helped NSCs move to damaged areas, turning into nerve and support cells and helping to repair long-term nerve injuries [97]. FCS from various sea cucumbers also helps to reduce inflammation and tissue damage by modifying the expression of important genes, including *NF-ĸb* (nuclear factor kappa-light-chain-enhancer of activated B cells), *TNFα* (tumor necrosis factor alpha), *iNOS* (inducible nitric oxide synthase), and *COX-2* (cyclooxygenase-2) [32].

### 3.4. Phenolic Compound

The body parts, including the body wall, tentacles, and viscera, of sea cucumbers all contain substantial amounts of phenolics with potent antioxidant properties [85]. The amount of phenolic compounds and their antioxidant properties vary depending on factors such as species, habitat, food habits, and harvesting period [98]. Several classes of phenolics may be distinguished from sea cucumbers, including phenolic acids, flavonoids, tannins, stilbenes, lignans, and coumarins [99]. The most common phenolic compounds found in sea cucumbers are chlorogenic acid (up to 93% by weight), gallic acid, *p*-coumaric acid, protocatechuic acid, ferulic acid, ellagic acid, cinnamic acid, catechin, rutin, quercetin, and pyrogallol [85], while the ascorbic acid content is minimal [100,101,102]. The antioxidant properties of sea cucumber phenolics have been studied by multiple researchers, which may have greater potential in neuroprotection by reducing ROS. Hossain et al. (2022) reported that around 23 phenolic compounds were isolated from the *C. frondosa*, containing mostly phenolic acids and flavonoids. These compounds showed antioxidant properties along with anti-tyrosinase and antiglycation properties, and inhibitory activities against low-density lipoprotein (LDL) cholesterol oxidation and DNA damage [37]. Again, 12 phenolic compounds were extracted from the aqueous extract of *H. tubulosa*, comprising mainly flavonoids and phenolic acids, showing high antioxidant activities. The total phenolic and flavonoid contents of AEs were reported to correlate with their antioxidant activity values [103].

The synergistic effect of these phenolic compounds with other bioactives in the in vivo *C. elegans* model, to treat diseases like AD and PD, suggested potential for natural preventive and therapeutic agents for neurorestoration [44,104,105]. The high radical scavenging capability (13.14 ± 2.17%) of the ethanolic extracts and fractions of *H. atra* demonstrated strong primary antioxidants, making them suitable for usage in the food and pharmaceutical sectors, as well as being a natural antioxidant that can be refined [106].

### 3.5. Fatty Acid and Phospholipid

Fatty acids in sea cucumbers can effectively improve impaired learning and memory functions related to aging and NDs [107,108,109]. Wang et al. (2020) found different phospholipid (PL) classes, along with an ether-PL sub-class from six different sea cucumbers by using a Normal Phase Liquid Chromatography–Triple-Quadrupole-Time-of-Flight Mass Spectrometry/Mass Spectrometry (NPLC-Triple-TOF-MS/MS) method [110]. The highest PL levels were rich in ether-phospholipids, which were obtained from the species *C. frondosa* (8.05 μmol/g) and rare phosphonoethanolamines were found for the first time in sea cucumbers [110]. Ermolenko et al. (2022) revealed the phospholipid profile of *A. japonicus,* analyzing the major structural PL glycerophosphoethanolamines (PEs), glycerophosphocholines (PCs), glycerophosphoserines (PSs), and glycerophosphoinositols (PIs) in tissues of wild and cultured sea cucumbers, mentioning that a diet with ω-3 polyunsaturated fatty acids (PUFAs) influences the PL profile, enhancing nutritional properties [111]. For example, eicosapentaenoic acid-enriched phospholipids (EPA-PLs) from sea cucumber, *C. frondosa*, help to improve Aβ-induced cognitive deficiency in a similar mechanism to docosahexaenoic acid phospholipids (DHA-PLs) in rats [107]. Moreover, in the PD mice model, EPA-PL improved behavioral deficiency by suppressing oxidative stress and apoptosis, thereby alleviating the loss of DA neurons via the mitochondria-mediated pathway and mitogen-activated protein kinase pathway [36]. Che et al. (2018) found that the neuroprotective effects of DHA/EPA-PLs depend on the molecular form, as eicosapentaenoic acid phosphatidylserine (EPA-PS) and docosahexaenoic acid phosphatidylserine (DHA-PS) could effectively protect PC12 from apoptosis [109]. Another study reported by Zhou et al. (2016) stated that phosphatidylcholine (PC) from sea cucumber *A. molpadioides* showed positive results in treating scopolamine-induced hippocampus impairment [108]. Due to higher level of EPA and DHA in PC, it showed better improvement, suppressing the malondialdehyde (MDA) level (28.80%) and monoamine oxidase (MAO) (33.64%) activity, and simultaneously increasing SOD (95.53 U/mg·prot.) activity [108]. Another saturated medium-chain fatty acid, decanoic acid, was isolated from *H. leucospilota* by Sanguanphun et al. (2022), which exhibited effectiveness against *C. elegans* PD models by inhibiting neurodegeneration [112].

### 3.6. Cerebrosides

Cerebrosides (Cers) are neutral substances that are vital to brain function. They are made up of ceramide (sphingosine and FA) and a monosaccharide that is connected to the C1 of esfingol by a β-glycosidic link. The white matter of the brain and the myelin sheaths surrounding nerves are rich in cerebrosides, while the cell membranes of other tissues contain trace amounts of these substances [113]. The level of sphingolipid (ceramides, cerebrosides, and gangliosides) in the brain rises with increased dietary intake of these compounds [114,115]. After consumption, it gets partially metabolized to glucosylated glucosylceramide (GlcCer) and sphingomyelin (SM), making it suitable to cross the blood–brain barrier (BBB) [116]. Several studies have been conducted on the protective role of cerebrosides in regulating brain functions. According to Li et al. (2019), Cers could significantly ameliorate Aβ1-42-induced cognitive deficiency from neuronal damage, suppressing the induced apoptosis [35]. Wu et al. (2013) showed that Cer from *A. molpadioides* protected brain cells from H_2_O_2_ and t-BHP-induced damage and increased the activity of a protective enzyme called SOD [117].

### 3.7. Other

Aside from the above groups of bioactives, other active compounds could be found in sea cucumber, which may exert a neuroprotective effect in pre-clinical and clinical models. For example, ethanol (ET), ethyl acetate (EA), butanol (BU), and aqueous (AQ) extract of *H. leucospilota* prevented the degeneration of DA neurons in the PD model, where terpenoids, steroids, saponins, and glycosides were identified from the EA extract [65]. Together with other phenolic compounds, the terpene friedelin, which was isolated from *H. scabra*, demonstrated possible antioxidant qualities [118].

A small cyclic ether, 2-butoxytetrahydrofuran (2-BTHF), was extracted from *H. scabra* and demonstrated its therapeutic potential against AD through the attenuation of Aβ aggregation in a transgenic *C. elegans* model [119]. Again, Jattujan et al. (2022) isolated five compounds: diterpene glycosides (holothuria A and B), palmitic acid, bis (2-ethylhexyl) phthalate (DEHP), and 2-butoxytetrahydrofuran (2-BTHF) from *H. scabra*. Among these five compounds, two of them, 2-BTHF and palmitic acid, demonstrated anti-aging activities [120]. Again, 14 carotenoids were identified from *C. frondosa japonica* using supercritical CO_2_ extraction, where cucumariaxanthin and canthaxanthin were abundant [121]. In addition, the fatty acid composition and carotenoids of 12 sea cucumbers revealed the cytotoxic activity of the carotenoids, and two particular fatty acid compounds (DHA and EPA) [122]. Previous studies demonstrated various biological activities and functions in different model systems, presented in Table 2.

## 4. Potential Therapeutic Applications

**Figure 4:**
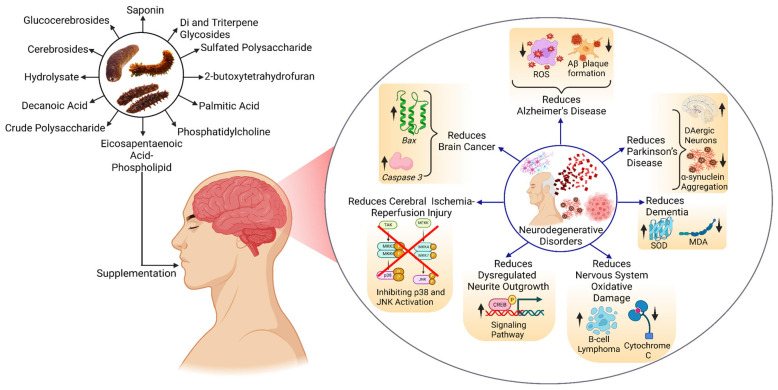
Effect of bioactive compounds on NDs. Abbreviations: CREB—cAMP Response Element-Binding Protein, MDA—Malondialdehyde, JNK—c-Jun N-terminal Kinase, SOD—Superoxide Dismutase, ROS—Reactive Oxygen Species. The figure was generated from the concept of Bonetto et al. (2025) [171] using Biorender.com (Agreement number: ZR28J5NO9Q).

The bioactive compounds from different sea cucumbers have shown positive results in combating NDs such as AD, PD, brain cancer, brain tumors, and other disorders. In treating AD, these compounds reduce ROS, which causes inflammation in the brain, and inhibit the formation of Aβ plaque, which is the main reason behind AD. These compounds also reduce the aggregation of alpha-synuclein (α-Syn), resulting in the improvement in the PD condition. Apart from these two important disorders, these compounds also play a role in treating brain cancer, reducing cerebral ischemia–reperfusion injury, dysregulating neurite outgrowth, and oxidative damage by modulating different signaling pathways or gene expression (Figure 4).

Despite having high potential for treating disease disorders, the neuroprotective effect is still in the preclinical research stage, with significant findings in AD and PD. Human clinical studies are very limited, and none of these focus on ND, limiting the implications or benefits of SCBs on human health [60,172]. Even though encouraging outcomes in preclinical studies using animal models and cell cultures are present, no bioactives as neurodrugs have yet advanced to the stage of clinical approval. In vivo research of SCBs is mostly focused on anti-tumor/anti-cancer activities, followed by lipid metabolism modulation and glucose metabolism management [173]. As a result, little is being explored regarding the mechanisms of action of these compounds.

### 4.1. Alzheimer’s Disease

Alzheimer’s disease, a progressive and incurable neurological condition, is diagnosed by a gradual breakdown of mental functions, leading to impaired decision making, communication, motor planning, and visual comprehension, which ultimately advances into dementia [174]. Numerous mechanisms, including mitochondrial breakdown, oxidative cellular stress, harmful accumulation of amyloid/tau (τ) proteins, and cholinergic deficits, are attributed to the etiology of AD [175]. A range of proposed theories have been suggested to explain the pathophysiology of AD, including the cholinergic hypothesis, Aβ deposition hypothesis, tau protein hypothesis, oxidative stress hypothesis, metal ion hypothesis, and neuroinflammation hypothesis [176], but the two primary ones are the hyperphosphorylation of the tau protein and the amyloid-β (Aβ) cascade. As AD progresses into advanced phases, it is linked to extensive Aβ plaques and tau aggregates as neurofibrillary tangles (NFTs), characterized by dementia [177,178].

Aβ is often a soluble short peptide that is created through a proteolytic process of α-secretase, β-secretase, and γ-secretase, cleaving the transmembrane protein, amyloid precursor protein (APP) [179]. However, the tau protein is mostly found in axons and is a member of the microtubule-associated protein family [180]. Along with the abnormal accumulation of Aβ, the hyperphosphorylation of the tau protein promotes the degeneration of neuronal stability. Depending on how much oligomerisation occurs, the imbalance between the synthesis and clearance of Aβ results in several kinds of harmful oligomers, including protofibrils, fibrils, and plaques [181]. Simultaneously, hyperphosphorylation of the tau protein creates an imbalance in the functions and stability of microtubules, resulting in neuronal death through the formation of an impaired double-helix fiber [182,183]. Research has revealed the functional interplay between Aβ and tau, linking them to the progressive deterioration of neuronal circuits and the impairment of cognitive functions observed in AD [184,185].

Other processes have been demonstrated to occur before the development of senile plaques and the deposition of NFTs, including oxidative stress, which is elevated in the aging brain [186,187,188]. The neuronal structural molecules are composed of a high proportion of PUFAs, which are highly susceptible to ROS and eventually lead to lipid peroxidation and subsequent apoptosis at the molecular level [189,190]. ROS generation in AD is both a cause and an effect of nuclear factor erythroid 2-related factor 2 (Nrf2) activation through the phosphatidylinositol 3-kinase (PI3K)/protein kinase B (AKT)/glycogen synthase kinase 3 beta (GSK3β), p62, p38, Mitogen-Activated Protein Kinase (MAPK)/Nuclear Factor kappa-light-chain-enhancer of activated B cell (NF-Κb) pathways, which are deeply associated with AD pathogenesis [191]. Additionally, the imbalance in metal ions (Fe, Cu, Zn, and Ca) triggers oxidative stress, evident by the elevated ROS and decreased levels of GSH, SOD, and antioxidant protein (ATOX). In addition to promoting Aβ overproduction by activating β- and γ-secretases and/or inhibiting α-secretase, oxidative stress can cause tau hyperphosphorylation by activating protein kinases (e.g., GSK-3β, cyclin-dependent kinase 5 (CDK5), MAPK, etc.) and/or inhibiting Protein Phosphatase 2A (PP2A) [192], suggesting one of the root causes of AD, supporting the metal ions hypothesis.

Given the importance of fundamental forebrain cholinergic neurones (BFCNs) in memory, learning, and cognitive function, the cholinergic hypothesis of AD states that acetylcholine (ACh) is necessary for cholinergic signal transduction associated with memory and learning [193]. It serves as a potent regulator and prerequisite for the complete expression of sensation-induced neurovascular coupling response [194]. Ach is synthesized by the enzyme choline acetyltransferase, whose catalytic activity depends on substrates like choline, acetyl-CoA, and ATP [176]. Along with the gradual and substantial decline in cognitive and behavioral abilities, Ach deficiency in AD patients is linked to abnormal cholinergic system activity that controls and encourages alterations in tau phosphorylation and APP metabolism, which results in neurotoxicity, neuroinflammation, and neuronal death [195,196].

Furthermore, elevated levels of inflammatory cytokines and related genes have also been linked to the onset of AD [197]. Under the neuroinflammatory theory, neuroinflammation, an innate host mechanism that helps shield and restore the brain’s normal structure and function from infections and injuries, is what drives the start of neurodegeneration [198]. It is distinguished by the activation of innate immune cells, permeable endothelium cells, microglia and astrocytes, and invading blood cells that result from mechanical or pharmacological damage to the BBB or brain structures [199,200]. Consequently, neuroinflammation induces the various immune system cells to produce and release inflammatory mediators, such as cytokines (IL-1β, IL-6, IL-18), chemokines (CCL1, CCL5, CXCL1), small-molecule messengers (prostaglandins and nitric oxide), and reactive oxygen [199]. These mediators can aggravate Aβ and τ pathologies [201].

Moreover, there are several components of the AlzPathway, which include the Aβ cleavage and degradation, apolipoprotein E (ApoE)-cholesterol pathway and the NFT accumulation, acetylcholine production, Wnt signaling pathway, the ubiquitin-mediated proteolysis, apoptosis, and calcium signaling pathway, the notch signaling pathway, the MAPK signaling pathway, the abnormal ceramide accumulation, reactive oxidation process, neurotrophin signaling pathway, the cell cycle, mammalian/mechanistic target of rapamycin (mTOR) signaling pathway, the lipid pathway, the insulin pathway, and the inflammation pathway [202]. Multiple comprehensive reviews reported that a clearly defined underlying cause of AD has not been identified yet [203,204], and no definitive treatment exists to date [205,206]. Nevertheless, recent studies are shifting towards the exploration of natural compounds with potential therapeutic efficacy [207,208].

### 4.2. Parkinson’s Disease

As an incurable neurodegenerative disease, PD comes after AD, with the second-highest incidence rate [209]. It is characterized by symptomatology involving both motor and non-motor aspects. The clinical manifestations of motor abnormalities are bradykinesia, hypokinesia, akinesia, hypomimia, hypophonia, drooling, swallowing issues, micrographia, stiffness (stiffness of limbs), unstable posture, and resting tremors [210]. However, nonmotor symptoms might express sooner and exert a substantial impact on quality of life. These symptoms include sadness, constipation, sleep difficulties, odd sensations, weariness, and dementia [211]. Multiple pathophysiological cascades underlie the progression of PD, which may include oxidative stress [212], mitochondrial damage [213], toxic exposure [214], aberrant protein folding and aggregation [215], dopamine neuron disruptions [216,217], the impairment of protein clearance pathways [218], autonomous cellular dysfunction [219], and the intracellular transmission of the prion-like protein [220]. Notably, one of the most prominent theories for PD is the buildup of misfolded proteins in intracellular regions [220,221].

The hallmark of PD is the loss of DA neurones in the midbrain’s substantia nigra pars compacta (SNpc), which is linked to Lewy bodies (LBs), a cytoplasmic inclusions that include insoluble misfolded alpha-synuclein (α-Syn) aggregates [222]. But PD also affects non-DA neurones and is typified by a more extensive pathology in other parts of the brain [223]. Based on the most pronounced mechanism of PD, LBs are spherical, eosinophilic, intraneuronal inclusions with a hyaline center and a pale peripheral halo made up of over 90 proteins [224]. The chronological formation of LBs and the subsequent deposition of α-Syn begins from the anterior olfactory nucleus and the dorsal motor nucleus of the glossopharyngeal and vagal nerves, and progresses to the brain stem, mesocortex, allocortex, and neocortex in later stages [225]. Based on the comprehensive review of the existing literature, Srinivasan et al. (2021) reported that the hereditary predisposition to early-onset PD is brought on by mutations in the genes for alpha-synuclein (SNCA), ATPase cation transporting 13A2 (ATP13A2), glucocerebrosidase (GBA), F-box protein 7 (FBX07), vacuolar protein sorting-associated protein 35 (VPS35), phospholipase A2, group VI (PLA2G6), DnaJ (Hsp40) homolog, subfamily C, member 6 (DNAJC6), Synaptojanin 1 (SYNJ1), Ubiquitin C-terminal hydrolase L1 (UCHL1), parkin (PRKN), leucine-rich repeat kinase 2 (LRRK2), PTEN-induced kinase 1 (PINK1), and DJ-1 which results in the abnormal protein conformations and interfere with the inherent cellular mechanistic ability to remove the misfolded proteins [226].

Despite the significant role of degenerative protein formation, dopamine persists as one of the crucial factors in the multifactorial etiology of PD progression. The functional role of dopamine in PD remains obscure due to its complex mechanism of action. The depletion of the dopamine level in the substantia nigra results in the disrupted coordination between the direct and indirect pathways, which is considered the leading cause of PD and reduced thalamocortical input [217,227]. Till now, clinically used anti-PD medications include monoamine oxidase B (MAO-B) inhibitors (selegiline, rasagiline, and safinamide), catechol-O-methyl transferase (COMT) inhibitors, dopamine precursors (levodopa and carbidopa), and dopamine agonists (pramipexole, ropinirole, rotigotine, and apomorphine) [228]. Due to the restricted penetration of exogenous DA and other catecholamines through the BBB, DA replacement therapy provides the foundation for the pharmacologic treatment of PD, which is mostly symptomatic [229].

### 4.3. Stroke and Ischemic Injuries

A stroke is a clinically observed condition presenting with acute and localized neurological impairment attributed to central nervous system vascular injury (infarction, hemorrhage) [230]. After a stroke, neurological functional abnormalities include hemiplegia, the loss of sensory and vibratory feeling, balance issues, verbal issues, ataxia, impaired reflexes, ptosis (of the eyelid), field of vision deficits, aphasia, apraxia, facial numbness or paraesthesia, which exert a negative influence on quality of life [231]. The underlying pathology of the stroke determines whether it is ischaemic or haemorrhagic [232]. Approximately 87% of strokes are attributed to the predominant subtype, ischemic stroke, with intracerebral haemorrhagic (ICH) stroke contributing 10%, while only 3% are subarachnoid hemorrhages (SAHs) [233].

Cerebral ischemia is brought about by cerebral artery blockage, which prevents blood flow to a particular region of the brain. This deprives neurons of oxygen and energy, which negatively impacts energy-dependent functions in neuronal cells [234,235]. Consequently, after ischemia–reperfusion, survivors are still at a high risk for neurological impairments, disability, and other repercussions [236]. Restoring blood flow to the ischaemic penumbra as soon as possible is a key component of the clinical care of ischaemic stroke in order to save damaged neurons [237]. A number of factors can contribute to ischaemic stroke, such as large-artery atherosclerosis (which accounts for 45% of all events), cardioembolism (15–30%), systemic hypoperfusion, penetrating artery disease, carotid dissection, hypercoagulability of genetic syndromes, or other unpredictable causes [238]. In terms of the source, time interval, location, and intensity of ischemia, as well as age and comorbidities, the neurological impairment and clinical presentation following an ischaemic stroke show significant variety [239].

Bioactive properties of sea cucumber, such as anti-atherosclerosis, anti-coagulation, and anti-inflammation, could be a major source of post-stroke management [32,123,153,170]. These active biomolecules may be used as a dietary supplement to delay the onset of illness.

### 4.4. Brain Cancer and Brain Tumors

Brain cancer represents a severe malignancy to the CNS, comprising primary tumors that originate within the neural tissue and metastatic secondary tumors that spread from extracranial cancers [240]. Over decades of scientific research, brain tumors have remained the most fatal form of cancer [241]. Additionally, the prevalence of brain tumors is rising in some populations, maybe as a result of improvements in systemic cancer therapy and survival or in the detection of primary brain tumors [242,243].

Tumors are not just collections of cancer cells; they are also a diverse collection of an extracellular matrix, secreted proteins, and resident and invading host cells [244]. A brain tumor in the patient may cause specific neurological symptoms such as headaches, seizures, aphasia, weakness, sensory loss, visual problems, and ataxia, or no symptoms if it happens accidentally [245].

Mounting evidence suggests that the microenvironment of the tumor accelerates the progression of cancer as a potent facilitator [246]. The three most common types of brain tumors are brain metastases, meningiomas, and gliomas or glioblastomas (GBMs) [245]. As outlined in the world health organization (WHO) classification system, brain tumors could be classified as either grade I or grade IV on a morphological, immunological, molecular, and genetic profiling basis [247], where GBMs, the most common and malignant tumor of the central nervous system, affect both children and adults with a slight predominance in males. Various malignant gliomas, which make up approximately 50.1% of all malignant brain tumors [248], are classified as a grade IV tumor based on histopathological features [249].

When a brain tumor (BT) develops in the cranium, the micro-vessels in the peritumoral areas are compressed, which reduces the cerebral blood flow (CBF) locally [250,251,252]. The term “blood–tumor barrier” (BTB) refers to another malfunction of the BBB that occurs during tumor growth [253] and is distinguished by the loss of astrocytic endfeet and neural connections, as well as an abnormal pericyte distribution [254]. Glioma cells that invade the body can physically push out astrocytic endfeet and damage the integrity of the BBB [255]. Although certain large and small molecules can pass through the BTB, it is not sufficiently permeable to permit the accumulation of substantial drug concentrations from the periphery inside the BT [256].

As an effective treatment approach, efforts have been concentrated on developing the next-generation targeted therapies or medications that could have superior BBB penetration capabilities. The several methods for enhancing medication transport across the BBB/BTB can be divided into two categories: those that are minimally invasive and those that are non-invasive. Despite not having reached their full potential, intrusive methods, which include direct access to the site of affliction, are presently undergoing significant optimization and improvement, with encouraging preclinical results [256]. These bioactive compounds have shown significant potential to fight against brain-oriented diseases with varying efficacy depending on the type of model and administered dosage (Table 3).

## 5. Neuroprotective Mechanism Involved

### 5.1. Alzheimer’s Disease

#### 5.1.1. In Lowering Oxidative Stress

Many studies have been performed to evaluate Aβ-induced cognitive dysfunction by observing learning and memory ability through different behavioral experiments [267,268,269]. The bioactive compounds from sea cucumber improve this cytotoxic condition by regulating the mitochondria-dependent apoptotic pathway [35,60,270]. The generation of Aβ in the brain is affected by oxidative stress, which leads to lipid peroxidation, protein oxidation, and DNA oxidation in the nervous system [271]. The mitochondrial respiratory chain is the main source of ROS-generated oxidative stress [272], and according to Che et al. (2017), cerebrosides from sea cucumber inhibited Aβ-induced cell apoptosis by the mitochondria-dependent apoptotic pathway. These compounds downregulated Caspase-9, cleaved Caspase-3, total Caspase-3, and *Bax*, and upregulated *Bcl*-2 protein in the target cell, leading to the breakdown of the membrane integrity of mitochondria, thus resulting in Aβ-induced cell apoptosis [270]. Caspase-9 initiates apoptosis in the cell, while Caspase-3 degrades neuronal components that are important for the brain, and *Bax* promotes mitochondrial membrane permeabilisation [273,274,275]. However, *Bcl*-2 is an anti-apoptotic protein that plays a role in stabilizing mitochondrial integrity and inhibiting *Bax* activity, thereby preventing cell death [276,277]. In the case of the progression of AD, higher levels of Caspase-9, cleaved Caspase-3, and Bax, and lower levels of Bcl-2 cause neurodegenerative problems. In a study on evaluating the impact of cerebroside on mice, the level of Caspase-9 and Caspase-3 was found to be decreasing from approximately 280% to 100% of Normal and approximately 240% to 120% of Normal, respectively, indicating the inhibition of Aβ-induced cell apoptosis [270].

Moreover, oxidative stress indicators like SOD, nitric oxide (NO), nitric oxide synthase (NOS), MDA, 8-hydroxy-2′-deoxyguanosine (8-OHdG), and 8-oxo-guanine (8-oxo-G) play vital roles in assessing the success of preventing Aβ-induced oxidative stress [270]. According to Che et al. (2017), cerebrosides were found to lower the level of SOD activity (from 40 U/mgprot to around 35 U/mgprot), MDA (from approximately 1.3 nmol/mgprot to around 1 nmol/mgprot), and the amount of NO (from 20 μmol/gprot to around 8 μmol/gprot), and elevated the content of NOS (from 10 μmol/gprot to around 12 μmol/gprot), 8-OhdG (from approximately 8 ng/mgprot to around 9 ng/mgprot), and 8-oxo-G (from approximately 43 pg/mgprot to around 52 pg/mgprot) in brain tissue, suggesting that these compounds can reverse cognitive impairment as well as reduce or stop the production of the Aβ protein in the brain [270].

#### 5.1.2. In Improving Synaptic Plasticity and Ameliorating Nerve Fiber Tangles

**Figure 5:**
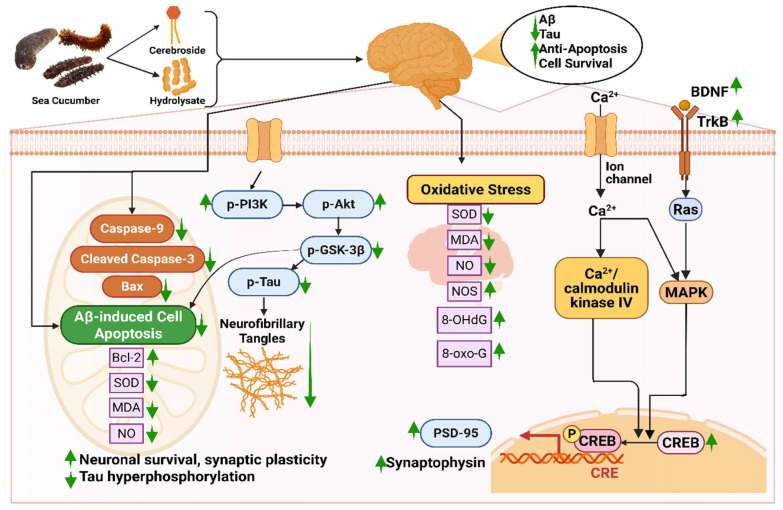
Neuroprotective mechanisms of sea cucumber-derived bioactive compounds in Alzheimer’s disease models. Abbreviations: 8-OHdG, 8-Hydroxy-2′-deoxyguanosine; 8-oxo-G, 8-oxo-guanine; Aβ, amyloid-beta; Bax, Bcl-2-associated X protein; Bcl-2, B-cell lymphoma 2; BDNF, brain-derived neurotrophic factor; CRE, cAMP response element; CREB, cAMP response element-binding protein; MDA, malondialdehyde; MAPK, mitogen-activated protein kinase; NO, nitric oxide; NOS, nitric oxide synthase; p-Akt, phosphorylated protein kinase; p-PI3K, phosphorylated phosphoinositide 3-kinase; p-Tau, Phosphorylated tau protein; PSD-95, postsynaptic density protein 95; Ras, renin-angiotensin system; SOD, superoxide dismutase; TrkB, tropomyosin receptor kinase B. The figure was generated from the concept of Li et al. (2019) [35] using Biorender.com (Agreement number: ZG28FPN3IU).

Bioactive compounds like hydrolysate, cerebrosides, or glucocerebrosides from sea cucumber reduce Aβ-induced cognitive deficiency by triggering the BDNF/TrkB/CREB pathway and escalating the expression of PSD-95 and synaptophysin [35]. Firstly, these compounds increase the amount of brain-derived neurotrophic factor (BDNF) protein in bioactive compound-injected mice [35,278]. BDNF plays a role in neuronal survival and growth, which later binds with specific tropomyosin kinase B (TrkB) receptors and elevates the TrkB level [279]. The binding of these two proteins elevates the level of cAMP response element-binding protein (CREB), which is essential for maintaining the activity of synapses [280]. A higher level of CREB improves the level of Postsynaptic density protein-95 (PSD-95) and synaptophysin, indicating improved synaptic plasticity [281]. Moreover, the bioactive compounds ameliorate nerve fiber tangles via the PI3K/Akt/GSK-3β pathway, in which levels of p-P13K and p-Akt are increased; on the contrary, p-Tau and p-GSK3β are decreased [35]. The protein kinases, phosphatidylinositol 3-kinase (PI3K) and phosphorylated Akt (p-Akt), help in cell survival and protection [282]. Interaction between these two kinases is the main reason behind neuronal survival, synaptic plasticity, and resistance to oxidative stress. Any form of dysregulation or breakdown in the PI3K/AKT signaling pathway results in Aβ accumulation, tau hyperphosphorylation, and mitochondrial dysfunction [282]. Again, phosphorylated tau protein (p-Tau) is a key biomarker in diagnosing AD that is responsible for the degeneration of neurons and synaptic impairment [283], whereas due to glycogen synthase kinase-3β (GSK-3β), hyperphosphorylation of the tau protein occurs, resulting in the formation of abnormal clumps of tau protein called neurofibrillary tangles (NFTs) (Figure 5) [284].

In a study by Li et al. (2019), the positive neuroprotective effect of sea cucumber-derived cerebrosides was studied in which levels of BDNF, p-CERB, and PSD95 were increased from approximately 1 to 1.2 folds, 1 to a 1.5 folds, and 1 to 1.1 folds, respectively, indicating enhanced neuronal survival, transcriptional activity, and synaptic integrity [35]. In the same study, the content of p-PI3K and p-Akt was found to upregulate from 1 to 1.1 folds to 1 to 1.25 folds, respectively. It was suggested that cerebrosides suppressed the phosphorylation of the tau protein by the PI3K/Akt/GSK3β pathway.

In another study, Gong et al. (2025) provided insights on how the gut–brain axis affects the gut microbiota in aging rats with cognitive impairment through the oral administration of sea cucumber hydrolysate (SCH). SCH restored gut microbiota homeostasis, balancing the *Bacillota/Bacteroidota* ratio and increasing beneficial taxa such as *Lachnospiraceae* and *Verrucomicrobiota*, which was also accompanied by enhancing cholinergic function, activating BDNF/TrkB signaling, and attenuating neuroinflammation mediated via inhibition of NF-κB and microglial activation. The study potentially addresses the neuroprotective effect of low-molecular-weight peptides containing key amino acids (Gly, Glu, Pro, Arg) to support neurotransmission, immune modulation, and BBB integrity [285].

### 5.2. Neuroprotective Mechanism Involved in Parkinson’s Disease

Different bioactive compounds from sea cucumber, such as extracts of *H. leucospilota*, *H. scabra*, *C. elegans* model, and *S. japonicus*, are being studied for treating PD [65,262,286]; however, the neuroprotective mechanism is yet to be discovered. According to Malaiwong et al. (2019), activation of the ubiquitin-proteasome system (UPS) is one of the mechanisms behind the success of *H. leucospilota* extract in having anti-Parkinson effects [65]. Ubiquitin in the UPS is a 76-amino-acid conserved protein that plays a role in controlling and assisting the protein denaturation process [287]. The ubiquitin binds with the targeted protein and, via catalytic reactions, breaks down the protein. For this reaction, three types of enzymes, such as E1 (ubiquitin-activating enzyme), E2 (ubiquitin-conjugating enzyme), and E3 (ubiquitin–protein ligase), are used [288,289]. Toxic intracellular proteins can accumulate due to abnormal protein stability, resulting in hampering neural homeostasis. Accumulation of the aggregated and misfolded brain protein, α-synuclein, is one of the main reasons behind PD-linked mutations of the α-synuclein gene, which can interfere with different cellular and molecular functions, thus inducing neurotoxicity [290].

Moreover, the generation of ROS inflicts damage on the substantia nigra of the brain via the peroxidative degradation of lipid, protein, and DNA oxidation [291]. Monoamine oxidase (MAO) activation, mitochondrial malfunction, alterations in the brain’s iron level, or even modifications to the antioxidant defense system appear to be the primary causes of this occurrence [292,293,294,295]. It has also been noted that NFTs with hyperphosphorylated tau protein are prevalent in PD brains [296,297]. Additionally, inflammatory cytokines, chemokines, GFAP, and nNOS are reported to be abundant in PD brains [298,299]. The corresponding evidence of the elevated expression of α-synuclein in enteric neurites parallels the intensity of intestinal wall inflammation, besides neuroinflammation [300,301].

**Figure 6:**
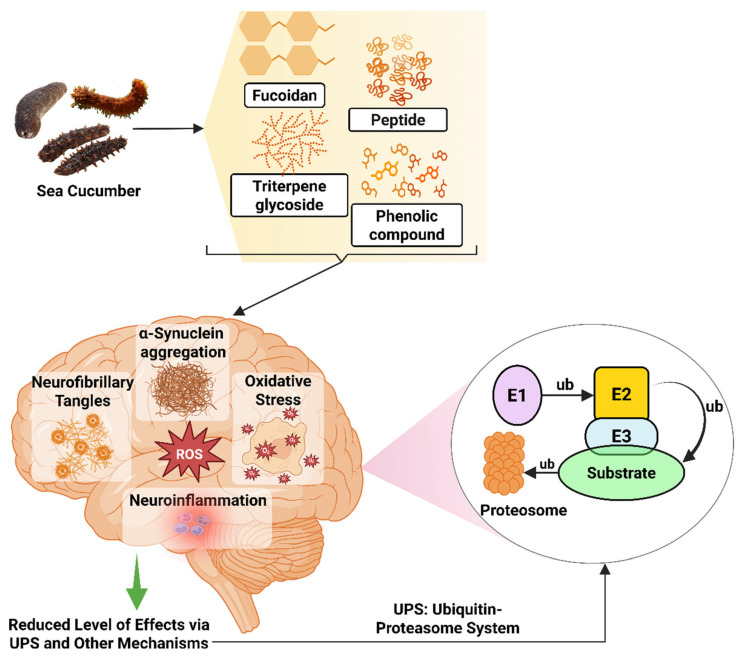
Neuroprotective effects of sea cucumber-derived bioactive compounds in PD models. E1, Ubiquitin-activating enzyme; E2, Ubiquitin-conjugating enzyme; E3, Ubiquitin ligase; ub, ubiquitin. The figure was generated from the concept of Lim and Tan (2007) [302] using Biorender.com (Agreement number: FH28FPNE87).

Studies have been carried out to understand how specific compounds from sea cucumbers provide neuroprotective effects in PD models. Diterpene glycosides extracted from *H. scabra* decrease α-synuclein accumulation and protect α-synuclein-mediated DA neuronal loss and its toxicities via *lgg-1* and *atg-7* in *C. elegans* PD model [261]. The ethanolic extract showed neurorestoration effects on maintaining the numbers of DA neurons and fibers in both substantia nigra pars compacta (SNpc) and striatum in both mice and cellular models induced by 1-methyl-4-phenyl-1,2,3,6-tetrahydropyridine (MPTP), as determined by a grid walk test, in a cellular model of PD [104]. Another study reported by Sanguanphun et al. (2022) revealed that decanoic acid isolated from *H. leucospilota* exerts an anti-Parkinson effect in *C. elegans* PD models by partly modulating the IIS/DAF-16 pathway, attenuating DA neurodegeneration, improving DA-dependent behaviors, and reducing oxidative stress in 6-OHDA-induced *C. elegans* (Figure 6) [112].

The regulation of cognitive health via gut microbiome alterations has been brought on by multiple studies [303,304,305]. Imbalance or dysbiosis of the intestinal microbiome is associated with functional changes in the CNS, facilitated by microbial metabolites and bidirectional interactions with the nervous, immune, and endocrine system along the gut–brain axis [306,307]. Some of the bioactive compounds from sea cucumber have been found to play a role in reducing this dysbiosis. For instance, FCS from sea cucumbers reduces inflammation linked to dysbiosis by lowering *Staphylococcus* levels through gut–brain axis regulation, which could mitigate PD symptoms. Dysbiosis can be measured by assessing the ratio of the phylum *Firmicutes* to the *Bacteroidota*. A study on the effect of FCS in treating PD showed a greater percentage of *Bacteroidota* and fewer *Firmicutes*, reducing the dysbiosis and resulting in improved gut and brain health [308].

## 6. Preclinical and Clinical Evidence

### 6.1. Alzheimer’s Disease

Bioactive compounds from sea cucumber have shown a promising effect in several AD models targeting specific disease-related pathways. In a transgenic *C. elegans* model of AD, Frondoside A considerably postponed the worm paralysis brought on by Aβ aggregation, reducing the level of small oligomeric forms, the most toxic species of Aβ, and subsequently turning down ROS production at a low dose of 1 µM [66]. Hydrolysates from sea cucumber (*S. japonicus*) exhibited higher β-secretase inhibitory activity, which serves as a key regulator in the amyloidogenic pathway and catalyzes the formation of neurotoxic Aβ peptide, and the reduction in BACE, sAPPβ, β-amyloid, p-JNK, and p-p38 in SH-SY5Y cells [258]. Sea cucumber peptides (SCP) derived from *A. leucoprocta* improved cognitive dysfunction in D-gal-induced aging mice. SCP plays role in releasing GABA by activating the GABA_B_R/cAMP/PKA/CREB pathway and alleviate neuronal and oxidative stress damage, subsequently ameliorating cognitive dysfunction [83]. Sulfated polysaccharides from *C. frondosa* disrupt preformed Aβ40 fibrils by disassembling mature fibrils [94].

A study described by Li et al. (2019) found that Cer from the body wall of the sea cucumber (*A. molpadioides*) ameliorated Aβ1-42-induced neuronal damage and suppressed induced apoptosis by decreasing the *Bax*/*Bcl*-2 ratio [35]. Additionally, Cer enhanced the expressions of PSD-95 and synaptophysin by activating the BDNF/TrkB/CREB signaling pathway, thereby ameliorating Aβ1-42-induced synaptic dysfunction. Furthermore, Cer attenuated Aβ1-42-induced tau hyperphosphorylation by activating the PI3K/Akt/GSK3β signaling pathway in male SD rats as an Alzheimer’s disease model. Additionally, 2-BTHF, a cyclic ether from *H. scabra*, has been suggested as a possible treatment for AD at 1 µg/mL and may shield *C. elegans* from Aβ toxicity by inhibiting its aggregation through an HSF-1-regulated autophagic mechanism [119].

Furthermore, in a study on the in vivo model of AD, dietary glucocerebrosides (SCGs) from sea cucumber (*C. frondosa*) influenced fatty acid hydroxylation or exosome-mediated Aβ clearance, resulting in a 30.7% reduction in hippocampus Aβ42 when compared to untreated AD animals. Fatty acid hydroxylation and exosome-mediated Aβ clearance are thought to be modulated to produce this effect, although there is no concrete evidence that SCGs are connected to any particular enzymatic pathways, such as β-secretase inhibition. SCGs may also help preserve neurones by maintaining myelin integrity, controlling ceramide/sulfatide ratios and lipid remodeling in the brain’s sphingolipid profile [259]. The study described by Li et al. (2019) found that cerebrosides from the body wall of the sea cucumber (*A. molpadioides*) ameliorated Aβ1-42-induced neuronal damage and suppressed apoptosis by decreasing the *Bax*/*Bcl*-2 ratio. Additionally, Cer enhanced the levels of PSD-95 and synaptophysin by activating the BDNF/TrkB/CREB signaling pathway, thereby ameliorating Aβ1-42-induced synaptic dysfunction. In another study, it was found that Cer attenuated Aβ1-42-induced tau hyperphosphorylation by activating the PI3K/Akt/GSK3β signaling pathway in male AD-infected rats [35].

Another bioactive, Frondoside A (FA), has been proven to postpone the worm paralysis brought on by Aβ aggregation in a transgenic *C. elegans* model of AD and subsequently turns down ROS production at a low dose of 1 µM. The study also implies that FA may target the early stages of Aβ peptide formation, preventing the development of these neurotoxic species [66].

In addition, inhibition of β-secretase (BACE1) in the amyloidogenic pathway has been studied to reduce pathogenic Aβ formation. According to Ma et al. (2021), the BACE1 inhibitory activity of *S. japonicus* crude polysaccharide (IC50: 16.13 µg/mL) was higher than trypsin hydrolysate (IC50: 93.59 µg/mL) due to the interference of sulfate-rich structural characteristics with the interactions between the enzyme and the substrate [258].

### 6.2. Parkinson’s Disease

As the bioactives from the body part of the sea cucumber possess anti-PD therapeutic potentials, multiple studies have been conducted to explore the pharmacokinetics and pharmacodynamic effects of these compounds. Using the toxin 6-OHDA to damage the nigrostriatal pathway and cause motor impairment, the striatal injection is one of the most commonly used animal models of PD [309].

Together with other bioactive substances from the body wall and Cuvierian tubule of *H. leucospilota*, saponin-rich extract improved DA neuronal function in food-sensing behavior and reduced α-synuclein aggregation, which in turn promoted the neuroprotection and regeneration of DA neurons in 6-OHDA-treated *C. elegans* PD models by downregulating the apoptosis gene (*egl-1*) and upregulating the genes that govern DA-synthesis (*cat-2*) and free-radical scavenging (*sod-3*) [65]. Chalorak et al. (2018) elucidated that triterpene glycosides and phenolic substances found in *H. scabra* extracts considerably reduced the degeneration of DA neurones in the BZ555 strain caused by the selective catecholamine neurotoxin 6-hydroxydopamine (6-OHDA) while improving food-sensing behavior, extending longevity, decreasing α-synuclein aggregation, and restoring lipid content in NL5901 [44]. Saponin from *C. frondosa* in the *C. elegans* PD model was tested for toxicity and optimal concentration by food clearance assay, and used to treat 6-OHDA-induced BZ555 strain and transgenic α-synuclein NL5901 strains in *C. elegans*. Treatment with the extract significantly attenuated DA neurodegeneration induced by 6-OHDA in the BZ555 strain, improved the basal slowing rate, and prolonged lifespan in the 6-OHDA-induced wild-type strain with the downregulation of the apoptosis mediators, *egl-1* and *ced-3*, and the upregulation of *sod-3* and *cat-2*. Interestingly, only FA reduced α-synuclein aggregation, rescued lifespan in NL5901, and upregulated the protein degradation regulators, including *ubh-4*, *hsf-1*, *hsp-16.1*, and *hsp-16.2* [260].

EPA-PL was extracted from the sea cucumber (*C. frondosa*) and applied to PD mice induced by MPTP, which improved behavioral deficiency by suppressing oxidative stress and apoptosis, thereby alleviating the loss of DA neurons via the mitochondria–mediated pathway and mitogen-activated protein kinase pathway [36]. At 100 μM, 2-BTHF significantly decreased αsynuclein accumulation and DA neurodegeneration, as molecular docking revealed that 2-BTHF may bind to HSF-1 and DAF-16 transcription factors. It also increased the mRNA transcripts of genes that encode proteins involved in proteostasis, such as the ubiquitination/SUMOylation-related *ubc-9* gene, the autophagy-related genes *atg-7* and *lgg-1*, and the molecular chaperones *hsp-16.2* and *hsp-16.49*. However, PPAR signaling pathways that mediated fatty acid metabolism were upregulated, according to transcriptome profiling. The 2-BTHF improved gcs-1-mediated glutathione production, increased the *fat-7* gene, and markedly reversed lipid accumulation in the *C. elegans* PD model [266]. These results suggested that sea cucumber extracts and their active ingredient compounds may have anti-PD potential.

Chalorak et al., in 2021, demonstrated that HSEA-P1 and HSEA-P2, diterpene glycosides from *H. scabra*, attenuated α-synuclein accumulation with the protection of DA neurons, and restored dopamine-dependent behaviors in *C. elegans* models, specifically through autophagy-related genes (*lgg-1* and *atg-7*). This occurred due to the mechanism involved in enhancing protein clearance as a critical pathway for α-synuclein degradation, while upregulating genes like *bec-1*, *lgg-1*, and *atg-7* [261]. This inconsistency suggests that HSEA compounds target a specific subset of the autophagy pathway, potentially omitting unc-51-mediated vesicle nucleation [310] or atg-18-mediated lysosomal fusion [311].

According to Promtang et al. (2024), 2-BTHF from *H. scabra* substantially lowered oxidative stress markers and α-synuclein accumulation in the muscle cells and DA neurones of the transgenic *C. elegans* model of the PD brain. The antioxidative capabilities of this compound are expressed by promoting lipid restoration and increasing glutathione production. However, 2-BTHF demonstrated relatively minor, non-significant effects on DA neurones (UA44), indicating cell-type-specific action or variable sensitivity, while significantly decreasing monomeric α-synuclein in muscle cells (NL5901) [266]. Additionally, this study suggests that the small molecule 2-BTHF may be able to cross the BBB by diffusion without restriction due to its low molecular weight and the formation of fewer than eight hydrogen bonds. These results imply that 2-BTHF may interact with the transcription factors HSF-1 and DAF-16 [312] and also correspond with the evidence of anti-aging properties of 2-BTHF [120]. However, there is still a lack of evaluation of the effectiveness of the drug due to a lack of a physiologically intact BBB [313].

Another bioactive compound, Frondoside A (FA), demonstrated neurorescue effects on DA neurodegeneration. In worms with α-synuclein overexpression, it significantly increased the mRNA levels of protein degradation regulators (*hsp-1*, *ubh-4*, *hsp-16.1*, *hsp-16.2*), indicating protein degradation pathways, an enhanced ubiquitin-proteasome system (UPS), and heat shock proteins (HSPs). Instead of using direct experimental exposure in the *C. elegans* model, this study claims that FA’s sulfate and acetyl groups are responsible for its better efficacy on broader structure–function relationships [260]. Again, two saturated fatty acids, decanoic acid (C10:0) and palmitic acid (C16:0), employ different but converging pathways for reducing oxidative stress, α-synuclein aggregation, and DA neurodegeneration in the *C. elegans* PD model. In that study, palmitic acid regulated autophagy and lipid metabolism, whereas decanoic acid was associated with conferring neuroprotection by activating the insulin/IGF-1 signaling (IIS) pathway, specifically through the nuclear translocation of the transcription factor DAF-16. Although both FAs exhibited non-linear dosage responses, palmitic acid’s dual autophagy-lipid regulation could render it more effective in synucleinopathies [112,265].

### 6.3. Stroke and Ischemic Injuries

As a leading cause of stroke globally, atherosclerosis in the major intracranial arteries induces the physiological regulation of blood flow that results in considerable luminal stenosis as well as modest wall thickening [314]. Recent studies suggest therapeutic applications against the arterial plaque formations that eventually lead to stroke. In a comprehensive review, Wang et al. (2022) reported that naturally occurring active compounds could inhibit atherosclerotic plaque formation within the arteries that supply blood to the brain by regulating autophagy [315]. Additionally, Hahn and Hill (2015) stated that patients with smaller infarcts seem to benefit from anticoagulation within two weeks following an acute stroke, which also helps to avoid the early recurrence of infarction [316]. Multiple active biomolecules of different ranges have been characterized and explored from different species of sea cucumber to find out the subsequent anticoagulant properties, such as FCSs [86,146,168], sulfated fucan [150], and fucosylated glycosaminoglycan [165]. A potential anticoagulant action was demonstrated by the glycosaminoglycan that was isolated from *A. japonicus*, where anticoagulant activity was nearly identical to that of heparin at the same quantity, below 170 µg/mL [123].

By inhibiting the activation of MAPKs, another bioactive sulfated polysaccharide from *S. japonicus* may be able to significantly reduce cell apoptosis brought on by Na_2_S_2_O_4_-induced hypoxia/reoxygenation (H/R) injury. This could lead to a significant decrease in the *Bax*/*Bcl*-2 ratio, cleaved *caspase-3*/*caspase-3*, p53 phosphorylation, and cytochrome c release, making it a potential medication to prevent or treat cerebral ischemia–reperfusion injury [263].

### 6.4. Brain Cancer and Brain Tumors

There is a lack of research on the therapeutic potential of sea cucumber as an anti-tumor or anti-cancer agent for brain tumors but their antioxidative nature and chemopreventive potentials for other organs or tissues have been studied extensively [88,126,139]. However, a few studies have shown the positive effect of sea cucumber’s bioactives in the PC12 cell line to reveal their potential as anti-tumor and anti-cancer agents. In a study by Che et al. (2018), it was found that the neuroprotective effects of DHA/EPA-PLs depend on the molecular form, as EPA-PS and DHA-PS (phosphatidylserine) could downregulate the messenger RNA level of *caspase-3*, *caspase-9*, and *Bax*, and upregulate *Bcl*-2 at the protein level [317]. This modulation protects PC12 cells from oxidative stress and prevents mitochondrial-mediated apoptosis and perhaps helps in crossing the BBB [109].

Additionally, the anti-cancer efficacy of ethyl acetate extract from *H. scabra* body wall has been shown on human GBM cell lines (A172 and U87MG) through the mitochondria-mediated pathway [143]. But in this study, the BBB permeability of the compounds remains unaddressed, which is a critical gap for GBM therapeutics [318].

## 7. Challenges and Future Outlook

Preclinical research has demonstrated the positive impact of using bioactive compounds extracted from sea cucumbers to address and control brain-related disorders. However, there are still some research gaps that are inhibiting the bringing of these advances to patients and the use of these marine natural products in medicine; such challenges must first be tackled. Several research studies have been conducted on evaluating the role of some common bioactive compounds extracted from sea cucumbers in fighting brain-oriented diseases. Future studies should include the isolation, identification, and chemical and biological characterization of new bioactive compounds such as alkaloids, antioxidant phenolics, functional peptides, and other health-promoting components using advanced methods [319].

Inadequate knowledge is available about the role of bioactive peptides from sea cucumber in molecular mechanisms as potential nutraceutical agents for memory impairment using proteomics technology. Integrated omics tool, along with molecular docking, could be used in the future, as proteomics technology offers a promising technique for studying molecular mechanisms through large-scale protein analysis [79,93]. In addition, more studies should be performed to understand the exact mechanism of action, metabolism, distribution, and transport of the existing and newly found bioactive compounds from sea cucumbers in the central nervous system. Comprehensive studies should be performed to evaluate the safety of the compounds through proper and long-term clinical trials to observe the interaction of the compounds with the food matrix and movement in the digestive system. The molecular and cellular mechanisms will reveal information about the impact on neuroinflammation and synaptic plasticity, the microbiota–gut–brain axis, the interactive pathway, and the mechanism of action [45].

Different kinds of bioactive compounds are extracted from sea cucumbers; however, knowledge of the exact mechanisms of action and optimal dosages for these compounds in supporting brain health is limited. Again, some of the compounds, like holothurians, a type of triterpene glycoside, can be toxic for human health when applied at a high concentration, causing skin irritation or other adverse effects [27]. Moreover, only a few neurotransmitter-based drugs are approved by the FDA, among which some of the drugs possess a serious negative impact and show deteriorated conditions after 12 months of administration [320]. Even though bioactive compounds from sea cucumber have high potential in treating NDs, the ability to cross the blood–brain barrier (BBB) remains unclear, which is essential to be acknowledged as neurotransmitter-based drugs. To attain a successful transition to first-in-human (FIH) trials, sponsors must strategically coordinate an approach across a variety of disciplines, such as toxicological assessment, biomarker assessment, pharmacokinetic studies, and global compliance etc., all of which must be seamlessly incorporated.

Moreover, many studies on the positive impact of sea cucumbers in inhibiting the aggregation of Aβ peptides have been conducted in in vivo and in vitro models. However, human clinical trials have not been performed to validate these findings and investigate their potential therapeutic applications [60]. Due to their efficacy and potential contribution to the nutraceutical sector and logical dosage recommendations, marine pharmaceuticals have sparked growing attention in recent decades. Many of the naturally occurring compounds, like bioactive compounds from sea cucumbers, are of tremendous interest for prospective medication development as well as ingredients for novel leads and commercially successful products for industrial purposes, particularly medicines, agrochemicals, functional foods, and nutraceuticals [321]. However, in order to use the compounds for treating brain health, more long-term clinical studies need to be performed. Comprehensive human studies must be conducted, taking into account dosage, safety, and long-term efficacy. Except for such trials, the medicinal potential of sea cucumber-derived chemicals remains uncertain, despite promising bioactivities in laboratory conditions.

## 8. Conclusions

Neurodegenerative brain disorders are complex, for which new and multidirectional treatments are needed, and sea cucumber bioactives have shown good potential in this regard. The promising effect of bioactive compounds extracted from sea cucumbers in health management is yet to be fully explored. Potential in vivo studies, along with in vitro studies that describe the molecular- and cellular-level mechanisms, may increase the utilization of these highly nutritional marine species for the development of highly efficacious therapeutics. Interdisciplinary collaboration among marine biology for optimized cultivation, neuropharmacology for multi-target delivery, and clinical medicine for early intervention should be performed to accelerate the translation of sea cucumber bioactives into therapies. Apart from these, the collaboration of the experts from bioengineering, toxicology studies, and the pharmaceutical industry is also crucial to increase the bioavailability and targeted delivery of these compounds. Incorporation of advanced omics technologies along with computational modeling can be carried out to gain deeper insights into the mode of action of these compounds, facilitating the rationality of clinical trials. Knowledge of marine biologists in the identification and sustainable extraction of these compounds and neuropharmacologists in formulating targeted delivery strategies for treating ND provides a critical foundation for exploring the therapeutic potential of sea cucumber-derived bioactives in the prevention and management of brain-related disorders. This review serves as a bridge between marine natural products and neuroscience. It highlights evidence-based research on sea cucumber-derived bioactives in the management of brain-related disorders, emphasizing their multimodal therapeutic potential. The study also underscores the urgent need to elucidate the exact mechanisms of action to prevent neurodegeneration, improve prognostic outcomes, and reduce systemic side effects. The bioactive ingredients from sea cucumbers could greatly benefit from better use in protecting the nervous system and boosting brain function. Harnessing the therapeutic potential of sea cucumber bioactives could pave the way for novel, nature-based therapies in the prevention and management of brain-related illnesses.

## Figures and Tables

**Table 1:** Keyword and search phrase selection list.

Category	Terms
Organism	Sea cucumber, *Acaudina leucoprocta*, *Apostichopus japonicus*, *Cucumaria frondosa*, *Holothuria leucospilota*, *Holothuria edulis*, *Holothuria scabra*, *Holothuria tubulosa*, *Holothuria atra*, *Stichopus japonicus*, *Stichopus chloronotus*, *Stichopus variegatus*
Target Organ	Brain
Diseases	Neurodegenerative disorders, age-related brain disorder, Alzheimer’s disease (AD), Parkinson’s disease (PD), stroke, depression, tumors, Brain tumor, brain metastases
Pathologies	Toxic protein deposition in the brain, neurotoxicity, neuroinflammation, oxidative stress, Aβ40 aggregation, Lewy bodies, alpha-synuclein (α-Syn) aggregates, neurofibrillary tangles
Therapeutic areas	Therapeutic potentials, anti-tumor, anti-inflammatory, antioxidant, anti-cancer, and neuroprotective effects of DHA/EPA-PLs
Bioactive compounds	Sea cucumber (SC)-derived products, bioactive compounds, natural bioactive, peptides, phenols, triterpene glycosides, fucoidan, fucosylated chondroitin sulfate, cerebrosides, sphingoids, saponins, Frondoside A, Biopeptide, protein hydrolysates, sea cucumber peptides, sulfated fucan or fucoidan, non-sulfated polysaccharides, glucocerebrosides, 2-butoxytetrahydrofuran
Enzymes	Acetylcholinesterase (AChE) activity in the brain, and Dopaminergic (DA) neuronal function
Molecular targets	Amyloid precursor protein (APP), tau protein, dopamine
Hypotheses	Hypothesis related to Alzheimer’s disease, Aβ deposition hypothesis, tau protein hypothesis, oxidative stress
Barriers	Blood–brain barrier, blood-tumor-barrier (BTB)
Applications	Nutraceuticals and functional foods of sea cucumber
Brain Chemistry	Fatty acid and phospholipid, lipidomic profile of the brain
Research Evidence	Clinical and pre-clinical evidence, In vivo and In vitro studies on sea cucumber bioactives
Pathways	AlzPathway, PI3K/Akt/GSK3β pathway, daf-16/Forkhead box O (FOXO) pathway, BDNF/TrkB/CREB signaling pathway, DAF-16/FOXO insulin/IGF, and SKN-1/NRF2 signaling pathways

**Table 2:** Overall biological activities and functions of bioactive compounds derived from different sea cucumber species.

Species	Body Part	Bioactive Compounds	Type of Activity	Model	Function	Reference
*Stichopus japonicus*	Whole body	Amino acids, peptides	Antioxidative	In vitro	- α-chy-III (200 μg/mL) enhanced cell viability by 80% and decreased ROS formation by 70%	[72]
		Glycosaminoglycan	Anticoagulant		- Heparin exhibited almost the same anticoagulant activity as intact HG at concentrations below 170 µg/mL	[123]
*Stichopus variegatus*	Body wall, muscle	Fucosylated chondroitin sulfates (SvF1 and SvF2), Sulfated fucan (SvF3)	Cytotoxic activity	In vitro	- Effect of SvF3 on breast cancer cell line T-47D (23%) and MDA-MB-231 (26%) at 400 µg/mL dose.	[88]
		Non-Sulphated Triterpene Glycosides (Variegatusides)	Anti-fungal activity	In vitro	- Showed positive results against 6 fungal strains	[124]
		Phenolic compound, Carotenoid	Antioxidative	In vitro	- Antioxidant activity poorer than synthetic substances (DPPH and β-carotene bleaching assay: 6.31 mg/mL and 46.37%)	[125]
*Stichopus hermanii*	Whole body	Saponin, tannin, flavonoid, terpenoid, steroid	Anti-cancer and anti-microbial	In vitro	- HE on ovarian cancer cells showed a positive result	[126]
*Stichopus horrens*	Body wall	Phenolic compound	Antioxidative, cytotoxic activity	In vitro	- Aqueous extract (79.62%) showed strong antioxidant activity by effectively inhibiting β-carotene oxidation	[127]
*Holothuria leucospilota*	Whole body	Peptide	Antioxidant activity	-	- Hydrolysate protein < −30 kDa scavenges DPPH and FRAP (maximum and lowest at 5 and 2 mg/mL)	[73]
*Holothuria parva*	Tegument	Octadecanoic acid 2-hydroxy-1-(hydroxymethyl) ethyl ester, 2′, 6′ -Acetoxylidide 2-(diethylamino)-, 2-Palmitoylglycerol, 5-Cholesten3beta-ol formate, Diisooctyl phthalate, Hexadecanoic acid, and Arachidonic acid	Antiviral	In vitro	- Ethanolic extract at 46.5 μg/mL reduced HSV-1 viral titer in Vero cell culture	[128]
	Body wall	Glycerol, gluconic acid, ouabain, spectinomycin and capreomycin	Antibacterial	In vitro	- *E. coli* (150 mg/mL), *P. aeruginosa* (100 mg/mL), and *E. faecalis* (175 mg/mL) were inhibited	[129]
	_	Phenol and Flavonoid content,	Anti-cancer	In vitro	- After 24 h, 250 μg/mL extract reduced malignant B lymphocyte viability by 20%	[130]
	Body wall	α-cyanostilbene	Anti-cancer	In vitro	- Cell viability in hepatocytes from HCC rats decreased considerably at concentrations of 5, 10, 20, 40, and 100 μg/mL	[131]
*Holothuria forskali*	Digestive tract, gonad, muscle, body wall and respiratory tract	Phenolic compound, fat-soluble vitamins, sterol	Antibacterial	In vitro	- Ethyl acetate extracts of the digestive tract showed the most potent effect at 2 mg/mL against *E. coli*	[132]
	Whole body	Amino acid, Peptide	Antioxidative, anti-hypertensive	In vitro	- Showed 170 μmol TE/L antioxidant activity via DPPH assay. - Produces 58% less ACE-I inhibition than teprotide (IC50 = 83.63 μM)	[133]
*Holothuria mexicana*	Body wall	Fucosylated polysaccharide sulfate	Antithrombotic activities	In vitro	- At 150 g/mL, showed higher APTT activity than heparin	[134]
*Holothuria lessoni*	Body wall, Viscera	Saponin	Anti-fungal	In vitro	- anti-fungal activity against *F. pseudograminearum* and *R. solani* is strong at 40 μL	[53,57]
*Holothuria edulis*	Body wall	Phenolic compound	Antioxidative, cytotoxic activity	In vitro	- Aqueous extract showed a strong radical scavenging capacity on DPPH (IC50 = 2.03 μg/mL), then organic extract - Aqueous extract active against cancer cell line TE1 cells (IC50 = 78.0 μg/mL), A549 (IC50 = 132.0 μg/mL)	[127]
		Sulfated polysaccharides (sulfated fucans, fucosylated chondroitin sulfates, neutral glucan)	Anticoagulant	In vitro	- APTT (2.86 μg/mL) and TT (16.03 μg/mL) testing showed that FCSs have anticoagulant properties lower than heparin but higher than sulfated fucans	[86]
*Holothuria nobilis*	Body wall	Polysaccharide	Anticoagulant	In vitro	- APTT (4.33 μg/mL) and TT (22.66 μg/mL) testing showed that FCSs have anticoagulant properties lower than heparin but higher than sulfated fucans	[86]
*Holothuria polii*	Body wall	Fucosylated chondroitin sulfate	Anticoagulant	*-*	- FCSs have an estimated anticoagulant activity of 220 units/mg	[135]
		Non-sulfated hexaoside	Anti-tumor	In vitro	- Extract showed an inhibitory effect on cancer cell line MCF7 (IC50:17.4 mg/mL) and HCT116 (IC50:18 mg/mL)	[136]
		Fucoidan Bivittoside	Hematopoietic activity	In vivo	- At dose (2/8 mg/kg), increased WBCs and neutrophils, indicating normalization of CTX-induced leukopenia and neutropenia in mice	[137]
*Holothuria thomasi*	Body wall	Saponin	Anti-diabetic	In vivo	- Reduced level of serum glucose and increased serum insulin at 300 mg/kg B.wt in STZ-induced diabetic rats. - Reduce urea and creatinine levels	[138]
*Holothuria tubulosa*	Body wall	Triterpene glycosides	Anti-fouling activities	*_*	May inhibit photosystem Q(B) protein 1 in marine macro fouler (algae)	[38]
		Phenolic compound	Cytotoxic	In vitro	- Aqueous and methanolic extracts inhibited human cancer cells (A549, HeLa, PC-3, and MCF-7) at 200–1000 µg/mL	[139]
*Holothuria hilla*	Body wall	Fucosylated chondroitin sulfates	Anticoagulant	In vitro	- The concentration of 2APTT was 2.8 ± 0.1 μg/mL - Effectiveness was higher than enoxaparin but lower than heparin	[140]
*Holothuria fuscocinerea*	Body wall	Phenolic compound, Carotenoid	Antioxidative	In vitro	- Antioxidant activity poorer than synthetic substances (DPPH and β-carotene bleaching assay: 0.29 mg/mL and 45.75%) - Carotenoid concentration (33.64 ± 0.07 mol/g) was strongly correlated with antioxidant activity in sample extracts	[125]
*Holothuria atra*	Whole Body	Flavonoids, phenolic components, terpenoids, saponins, alkaloids	Cytotoxic, antiviral	In vitro	- Methanolic extract showed anti-proliferative activity against cervical cancer cell line Hela (IC50: 468 μg/mL), MCF-7 (IC50: 352 μg/mL) - Demonstrated antiviral activity against HSV-1 and HSV-2 with a 75% inhibition rate at 2.4 × 10^3^ pfu/mL	[141]
	Body wall	Phenolic compounds (chlorogenic acid, pyrogallol, rutin, coumaric acid, catechin, and ascorbic acid)	Antioxidative and hepatoprotective activity	In vitro, In vivo	- Extract showed great NO∙ scavenging activity (93.42% at 600 µg/mL) and low DPPH∙ activity (17.01% at 16.8 µg/mL) - Lipid peroxidation inhibition: 36.4% at 600 µg/mL	[100]
*Holothuria arenicola*	Body wall	Phenolic compound (chlorogenic acid, pyrogallol, rutin, coumaric acid	Anti-fibrotic	In vivo	- Oral extract (200 mg/k BW) decreased total conjugated and unconjugated bilirubin, serum aminotransferases, alkaline phosphatase, and in albino rats	[101]
Holothuria (Metriaty *Holothuria (Metriatyla) scabra*	Body wall	Sulfated triterpene glycoside (Scabraside D), triterpene glycoside	Anti-cancer,	In vivo, In vitro	- Impaired HuCCA cell viability and migration (IC50:12.8 ± 0.05 μg/mL at 24 h)	[142,143]
	Whole body	Friedelin, 3-Hydroxybenzaldehyde and 4-Hydroxybenzaldehyde	Antioxidative	In vitro	- DPPH free radical scavenging activity was observed in 3 compounds (EC50: 33.77 ± 0.24, 14.63 ± 0.01, 14.62 ± 0.01, and 14.68 ± 0.11 mg/mL	[118]
*Holothuria spinifera*	Whole body	Cerebrosides (spiniferosides A, B, C, and holospiniferoside) and cholesterol sulfate	Anti-tumor	In vitro	- Spiniferosides A, B, C, and cholesterol sulfate showed significant cytotoxic effects with IC50 values of 13.83, 8.13, 8.27, and 35.56 µM, respectively - Holospiniferoside showed antiproliferative effect (IC50: 20.6 µM)	[144,145]
	Body wall	Fucosylated chondroitin sulfates	Anticoagulant	In vitro	- APTT assay showed a two-fold increase in clot formation at 2.6 ± 0.1 μg/mL	[146]
*Holothuria impatiens*	Whole body	Triterpene tetraglycosides (25-Hydroxyfuscocineroside B, Fuscocineroside B, Pervicoside C, Holothurin A)	_	*_*	_	[147]
*Actinopyga lecanora*	Whole body	Peptide	Antioxidative activity, antihypertension	In vitro	- Showed ACE inhibitory action (1.50–2.54 mg/mL) - Alcalase protein hydrolysate exhibited the strongest DPPH radical-scavenging activity (IC50: 0.181 mg/mL)	[76]
*Apostichopus japonicas*	Body wall	Saponin (Holotoxin A1)	Anti-tumor	In vitro	- Showed antiproliferative effect strongest activity against HL-60 cells (IC50: 23.55 ± 3.40 µg/mL)	[148]
		Sulfated polysaccharide (Fucosylated chondroitin sulfate (FCS), sulfated fucan)	Anticoagulant	In vitro	- APTT (2.20 μg/mL) and TT (14.20 μg/mL) testing showed that FCSs have anticoagulant properties lower than heparin but higher than sulfated fucans	[86]
		Peptide	Anti-hyperuricemic and anti-inflammatory	In vivo	Reduces TLR4/MyD88/NF-κB signaling pathway activation, crucial for inflammatory response	[149]
*Acaudina leucoprocta*	Body wall	Sulfated fucan (AL1-1)	Anticoagulant	In vitro	- At 0–20 μg/mL, intrinsic anticoagulant activity on APTT was less than heparin, but did not extend PT or TT	[150]
		Peptide	Anti-aging, anti-hyperuricemic and anti-inflammatory	In vivo	Extended the lifetime of nematodes *Caenorhabditis elegans* by 31.46% - Reduces TLR4/MyD88/NF-κB signaling pathway activation, crucial for inflammatory response	[149,151]
		Peptide	Anti-fatigue	In vivo	- SCP-1 and SCP-2 enhanced exercise performance and reduced tiredness in mice by reducing oxidative stress and increasing mitochondrial function via NRF2 and AMPK pathways	[75]
*Acaudina molpadioides*	Body wall	Peptide	Antioxidative	In vitro	Microwave-assisted (300 W) hydrolysate had 100% more content and 109% more DPPH scavenging activity than non-irradiated sample	[34]
		Cerebroside	Hepatic adipopexis	In vivo	- Dietary SCC at the levels of 0.006% and 0.03% ameliorated the hepatic lipid accumulation in fatty liver rats	[152]
	Whole body	Fucosylated chondroitin sulfate	Anti-inflammatory	In vivo	Increased anti-inflammatory *IL-10* mRNA levels (114.18%), and reduced body weight gain (26.38%) by altering gut microbiota	[153]
		Fucoidan			- Inhibits pancreatic islet cell apoptosis by downregulating caspase 3 and 9	[154]
*Eupentacta fraudatrix*	Whole body	Triterpene glycoside Cucumariosides I2, H, A5, A6, B2 and B6	Cytotoxic, lysosomal	In vitro	- Cucumarioside H, A5, A6 showed moderate cytotoxicity against mouse Ehrlich cancer cells in ascites form (EC50 16.3, 17.3, 12.5 µg/mL) - Glycosides I2, A5, and B2 enhanced the lysosomal activity of macrophages by 15–17% at doses of 1–5 µg/mL	[155]
*Bohadschia cousteaui*	Body wall	Saponin (coustesides A, B, C, D, E, F, G, H, I and J)	Anti-fungal activity	In vitro	-Inhibited Candida albicans growth in 10 μL (1 mg/mL) stock solution.	[156]
*Colochirus quadrangularis*	_	Sulfated triterpene glycoside (coloquadranoside A), triterpene glycosides (philinopside A, B, E and pentactaside B)	Anti-tumor, immunomodulatory activity	In vitro, In vivo	- In vitro: Coloquadranoside A may reduce tumor development and aggressiveness by decreasing neovascularization via tyrosine kinase autophosphorylation pathway. - In vivo: Coloquadranoside A (5–50 mg/kg) inhibited tumor growth in S-180 and H22 homograft mice (TGI > 35%), and at 50–500 mg/kg enhanced CTX-induced macrophage clearance and phagocytosis in immunosuppressed mice	[157]
		Triterpene glycosides (quadrangularisosides A, A1, B, B1, B2, C, C1, D, D1–D4, and E	Cytotoxic,	In vitro	- Compounds 1–13 showed dose-dependent inhibition of HT-29 cells at concentrations of 0–20 μM, measured by MTS assay	[158]
*Neothyonidium magnum*	Whole body	Triterpene glycosides (magnumosides A1, A2, A3, A4, B1, B2, C1, C2, C4 and colochiroside B2)	Cytotoxic	In vitro	- Magnumoside A3, C1, C2, and C4 reduced Human Colorectal Adenocarcinoma DLD-1 Cell viability with IC50 values of 30.3, 34.3, 32.9, 37.1, and 33.9 μM - Magnumoside A3 and C1 reduced DLD-1 cancer cell colony size by 49% and 43% at 10 μM, respectively, and prevented spontaneous colony formation by 22% and 26%	[159]
*Isostichopus badionotus*	Body wall,	Peptide	Anti-hypertension	In vitro	- The 10 kDa fraction showed the highest angiotensin enzyme inhibitory factor (80.7%), with an IC50 of 83 µg/mL	[160]
	Whole body	Fucosylated chondroitin sulfate	Anti-metastatic, anti-hyperlipidemic	In vivo, In vitro	- In MTT assay, the compound reduced 95D cell viability (IC50: 369.8 µg/mL) - Showed 24.4% tumor growth reduction at 5 mg/kg in Lewis lung carcinoma mouse models	[161]
*Isostichopus fuscus*	Tentacles	Peptides	Antioxidative	In vitro	- The 3 kDa fraction had the highest ORAC value (0.92 ± 0.04 μmol TE/mg protein)	[71]
*Parastichopus tremulus*	Whole body	Amino acid, Peptide	Antioxidative	In vitro	- Antioxidant activity in ORAC assay is measured at 0.35 ± 0.05 TE/μg protein	[162]
*Athyonidium chilensis*	Body without gut	Saponin	Antibacterial, anti-fungal, cytotoxic	In vitro	- Showed anti-microbial efficacy against Gram-positive bacteria but not Gram-negative bacteria at 10 mg/mL concentration - Exhibited efficacy against fungus at conc. 10 mg/mL - Showed cytotoxic activity on N2A tumor cells (IC50: 77.34 ± 1.6 μg ml^−1^)	[163]
*Stichopus vastus*	Integument	Protein, Peptides	Antioxidative	In vitro	- Trypsin-hydrolyzed collagen hydrolysates reduced up to 71.3% of ABTS radicals	[164]
*Thelenota ananas*	Body wal	Fucosylated chondroitin sulfate	Anticoagulant, antiviral	In vitro	- Compound’s APTT increasing activities dropped from 125.8 to 14.8 U/mg as fucosylation degrees reduced from 100% to 34% - Strong thrombin inhibition (EC50 < 286 ng/mL) was observed with heparin cofactor II in the depolymerized molecule - Strong antiviral activity against T-20-resistant bacteria (EC50: 0.76–1.13 μg/mL)	[165,166,167,168]
	Body wall and alimentary canal	Saponin (Desulfated holothurin A)	Anti-cholesterol, anti-atherosclerosis	In vitro, in vivo	- Treatment with 1 μg/mL increased mRNA expressions of SR-BI, ABCA1, and ABCG1 by 2-fold, 2.4-fold, and 2.3-fold, respectively	[169,170]
*Paracaudina chilensis*	Body wall	Fucosylated chondroitin sulfates	Anticoagulant	In vitro	- Effectiveness was higher than enoxaparin but lower than heparin	[140]

Abbreviations: APTT: Activated Partial Thromboplastin Time (secondary measurement); ABCA1: ATP-Binding Cassette Transporter A1; ABCG1: ATP-Binding Cassette Transporter G1; ABTS: 2,2′-Azino-bis(3-ethylbenzothiazoline-6-sulfonic Acid); A549: Human Lung Carcinoma Cell Line; ALT: Alanine Aminotransferase; AMPK: AMP-Activated Protein Kinase; APTT: Activated Partial Thromboplastin Time; AST: Aspartate Aminotransferase; B16F10: Mouse Melanoma Cell Line; *Bcl*-xL: B-cell Lymphoma-extra large; BW: Body Weight; CAM: Complementary and Alternative Medicine; CTX: Cyclophosphamide; DAF-16/DAF-2/SOD-3/OLD1/PEPT-1 axis: A longevity-regulating signaling cascade in *C. elegans*; DPPH: 2,2-Diphenyl-1-picrylhydrazyl (radical scavenging assay); EC50: Half Maximal Effective Concentration; FFA: Free Fatty Acids; FAS: Fatty Acid Synthase; FRAP: Ferric Reducing Antioxidant Power; FCS: Fucosylated Chondroitin Sulfate; FXase: Factor Xase (Tenase complex); G6PDH: Glucose-6-Phosphate Dehydrogenase; GST: Glutathione S-Transferase; HCC: Hepatocellular Carcinoma; HeLa: Human Cervical Cancer Cell Line; Hep3B: Human Hepatoma Cell Line; HG: High Glucose; Hif-1α: Hypoxia-Inducible Factor 1-alpha; HL-60: Human Promyelocytic Leukemia Cell Line; HIV-2: Human Immunodeficiency Virus Type 2; HPa: Heparanase; HT-29: Human Colorectal Adenocarcinoma Cell Line; HSV: Herpes Simplex Virus; HuCCA: Human Cholangiocarcinoma Cell Line; JNK1/2: c-Jun N-terminal Kinases 1 and 2; LXR: Liver X Receptor; MCF-7: Human Breast Cancer Cell Line; ME: Malic Enzyme; MMP collapse: Mitochondrial Membrane Potential Collapse; MTS assay: Cell Viability Assay Using Tetrazolium Compound; MTT assay: 3-(4,5-dimethylthiazol-2-yl)-2,5-diphenyltetrazolium bromide Assay; N2A: Neuro-2A Neuroblastoma Cell Line; NO: Nitric Oxide; NRF2: Nuclear Factor Erythroid 2–Related Factor 2; OA-fed: Oleic Acid-fed (diet-induced model); ORAC: Oxygen Radical Absorbance Capacity; PC-3: Human Prostate Cancer Cell Line; PT: Prothrombin Time; ROS: Reactive Oxygen Species; SCC: Squamous Cell Carcinoma; SCP: Sea Cucumber Peptides; SREBP-1c: Sterol Regulatory Element-Binding Protein 1c; SR-BI: Scavenger Receptor Class B Type I; STZ-induced diabetic: Streptozotocin-Induced Diabetic Model; TG: Triglycerides; TLR4/MyD88/NF-κB signaling pathway: Toll-Like Receptor 4/Myeloid Differentiation Primary Response 88/Nuclear Factor kappa-light-chain-enhancer of activated B cell Pathway; TT: Thrombin Time; VEGF: Vascular Endothelial Growth Factor.

**Table 3:** List of bioactive compounds in sea cucumbers in the prevention of NDs using different models and their dose-responsive effects.

Compound	Disease	Model	Effect	Dose	Reference
Hydrolysate	Alzheimer’s disease	Aβ-mCherry cells model	- Aβ aggregation significantly decreased by 38.80%	0.5 mg/mL	[257]
Crude Polysaccharide	Alzheimer’s disease	SH-SY5Y cells model	- β-Secretase inhibitory activity (IC50, 16.13 ± 1.15 μg/mL) - Reduced BACE, Aβ, sAPPβ, p-p38, and p-JNK levels	-	[258]
Cerebrosides	Alzheimer’s disease	SAMP8 Mice and PC12 Cells	- Improved Aβ1-42-induced spatial memory impairment (target quadrant time: 45.5 s reduced to 17.1 s) - Activated the BDNF/TrkB/CREB and PI3K/Akt/GSK3β signaling pathway.	200 mg/kg	[35]
Glucocerebrosides	Alzheimer’s disease	SAMP8 mice model	- Aβ42 solubility in the brain part (hippocampus) decreased by 30.7% (target quadrant 32.6 ± 3.6% at 15.1 ± 2.0 s)	5 g/kg	[259]
Saponin (Frondoside A)	Parkinson’s Disease	*C. elegans*	- α-synuclein aggregation reduced by 20–30% - Extended mean lifespan 6.58% at 0.5 μM - Suppressed egl-1 and ced-3, sod-3, cat-2 - Upregulated *hsf-1*, *ubh-4*, *hsp-16.1*, and *hsp-16.2* mRNA at 1 μM	0.1–10 µM	[260]
Diterpene glycosides	Parkinson’s Disease	*C. elegans*	- α-synuclein aggregation reduced by 20–30% - DA neurons increased by 20% at day 5 - Upregulated *bec-1*, *lgg-1*, and *atg-7*	1–10 μg/mL	[261]
Frondoside A	Alzheimer’s disease	*C. elegans*	- Reduced Aβ oligomer (150 kDa) by 49.1% - ROS level reduced 46. 7%	1 μM	[66]
Triterpene glycosides	Brain cancer	A172 and U87MG cell lines	- Strong cytotoxic effect to A172 (IC50: 4.23 μg/mL and U87MG cells (IC50: 4.46 μg/mL) - Increased pro-apoptotic *Bax* and *caspase 3* - Decreased *Bcl*-2	1–10 µg/mL	[143]
Sulfated Polysaccharide	Parkinson’s Disease	SH-SY5Y cells	- ROS reduced by 60–70% - Suppressed *Bax*/*Bcl*-2, *cl-casp-9/casp-9*, and *cl-casp-3/casp-3* - Inhibited MAPK and Activated PI3K/Akt Signaling pathway	300 μg/mL	[262]
Sulfated Polysaccharide	Cerebral ischemia–reperfusion injury	PC12 cells	- SOD activity restored by 60–70% - Suppressed *Bax*/*Bcl*-2 ratio, cleaved *caspase-3*/*caspase-3*, p53 phosphorylation, and cytochrome c - Inhibited JNK1/2 and p38 MAPK activation	100–500 µg/mL	[263]
Cerebroside	Nervous system oxidative damage	PC12 cells	- SOD activity increased by 79%, - Increased the B-cell lymphoma 2 (*Bcl*-2) - Decreased Cytochrome c, *caspase3/9*	400 μg/mL	[117]
Glucocerebrosides	Dysregulated neurite outgrowth	PC12 cells	- Neurite-bearing cells increased by 60.7% - BDNF expression increased 3.02-fold	200 μg/mL	[264]
Phosphatidylcholine	Dementia	BALB/c mice	- MDA level decreased by 28.80%, MAO (33.64%) - Increased SOD activity (95.53 U/mg prot.) in white matter	5 g/kg	[108]
EPA-Pl	Alzheimer Disease	Mice	- Escape latency 22 s in Morris water maze test on day 5 - Increased SOD (62.0 ± 3.67 U/mg protein) activity - Reduced MDA 5.39 ± 0.98 nmol/mg protein, p-tau (0.12-fold), p-GSK-3β (0.52-fold) levels	300 mg/kg·day	[107]
2-butoxytetrahydrofuran (2-BTHF)	Alzheimer Disease	CL4176 and CL2006 *C. elegans* strain	- Reduced Aβ oligomer/actin CL4176 (24.5%) and CL2006 (28.4%) - Aβ deposition reduced CL2006 (76.5%), and ROS was reduced by 45.8% - Upregulated autophagy genes *bec-1*, *lgg-1*, *atg-7*, and *lmp-1*	1 µg/mL	[119]
Decanoic acid	Parkinson’s Disease	*C. elegans*	- Basal slowing rate decreased by 67.56% - ROS decreased from 239.58% to 101.81% - α-synuclein aggregation decreased by 19.10% - increased SOD-3 activity (107.42%), - Modulate IIS/DAF-16 pathway	5–25 μg/mL	[112]
Palmitic acid	Parkinson’s Disease	*Caenorhabditis elegans* PD models.	- α-synuclein aggregation was reduced by 24.28% - Reduced lipid deposition from 74.97% to 54.13% - Downregulated *fat-5*, and *fat-7* - Lifespan expanded by 25.97% - Upregulated *gst-10*, *gst-1*	5 µg/mL	[265]
EPA-PL	Parkinson’s Disease	Male C57BL/6J mice	- Increased the latent period on the rotarod test by 197% - Inhibited the activation of pro-apoptotic JNK and P38 MAPK pathway - Reduced the ratio of *Bax*/*Bcl*-2, *caspase-3/9*	20.25 (g/1000 g)	[36]
2-butoxytetrahydrofuran (2-BTHF)	Parkinson’s disease	*Caenorhabditis elegans* strain	- α-synuclein intensity reduced by 73.86 ± 3.31% - DA neurons increased by (85.21 ± 2.19%, - increased *hsp-16.2* and *hsp-16.49*, *ubc-9 gene*, and genes *atg-7* and *lgg-1*.	100 μM	[266]

Abbreviations: A172: Human Glioblastoma Cell Line; α-synuclein: Alpha-Synuclein; *atg-7*: Autophagy Related Gene 7; BALB/c: Albino Laboratory-Bred Mouse Strain; BACE: β-site APP-Cleaving Enzyme; *Bax*: *Bcl*-2-Associated X Protein; *Bcl*-2: B-cell Lymphoma 2; BDNF: Brain-Derived Neurotrophic Factor; *bec-1*: Beclin-1; *cat-2*: Catalase-2; *ced-3*: Cell Death Abnormality-3; CREB: cAMP Response Element-Binding Protein; DA: Dopaminergic; *egl-1*: Egg Laying Defective-1; *gst-1*: Glutathione S-Transferase 1; *gst-10*: Glutathione S-Transferase 10; GSK3β: Glycogen Synthase Kinase 3 Beta; *hsp*: Heat Shock Proteins; *hsp-16.1*: Heat Shock Protein 16.1; *hsp-16.2*: Heat Shock Protein 16.2; *hsf-1*: Heat Shock Factor 1; IIS/DAF: Insulin/IGF-1 Signaling/Dauer Formation Pathway; JNK1/2: c-Jun N-terminal Kinase 1/2; *lgg-1*: LGG-1 (LC3 homolog in *C. elegans*); *lmp-1*: Lysosomal Membrane Protein-1; MAPK: Mitogen-Activated Protein Kinase; p38 MAPK: p38 Mitogen-Activated Protein Kinase; p-GSK-3β: Phosphorylated Glycogen Synthase Kinase 3 Beta; p-JNK: Phosphorylated c-Jun N-terminal Kinase; p-p38: Phosphorylated p38 Mitogen-Activated Protein Kinase; p-tau: Phosphorylated Tau Protein; PC12: Pheochromocytoma Cell Line; PI3K: Phosphoinositide 3-Kinase; PI3K/Akt: Phosphoinositide 3-Kinase/Akt Pathway; PI3K/Akt/GSK3β: Phosphoinositide 3-Kinase/Protein Kinase B/Glycogen Synthase Kinase 3 Beta; SAMP8: Senescence-Accelerated Mouse-Prone 8; sAPPβ: Soluble Amyloid Precursor Protein Beta; SH-SY5Y: Human Neuroblastoma Cell Line; *sod-3*: Superoxide Dismutase-3; TrkB: Tropomyosin Receptor Kinase B; *ubh-4*: Ubiquitin Hydrolase 4; *ubc-9*: Ubiquitin-Conjugating Enzyme 9; U87MG: Human Glioblastoma Cell Line.
